# Calcium Binding by Arabinogalactan Polysaccharides Is Important for Normal Plant Development

**DOI:** 10.1105/tpc.20.00027

**Published:** 2020-08-06

**Authors:** Federico Lopez-Hernandez, Theodora Tryfona, Annalisa Rizza, Xiaolan L. Yu, Matthew O.B. Harris, Alex A.R. Webb, Toshihisa Kotake, Paul Dupree

**Affiliations:** a Department of Biochemistry, University of Cambridge, Cambridge CB2 1QW, United Kingdom; b Sainsbury Laboratory, University of Cambridge, Cambridge CB2 1LR, United Kingdom; c Department of Plant Sciences, University of Cambridge, Cambridge CB2 3EA, United Kingdom; d Graduate School of Science and Engineering, Saitama University, Saitama 338-8570, Japan

## Abstract

Arabinogalactan proteins (AGPs) are a family of plant extracellular proteoglycans involved in many physiological events. AGPs are often anchored to the extracellular side of the plasma membrane and are highly glycosylated with arabinogalactan (AG) polysaccharides, but the molecular function of this glycosylation remains largely unknown. The β-linked glucuronic acid (GlcA) residues in AG polysaccharides have been shown in vitro to bind to calcium in a pH-dependent manner. Here, we used Arabidopsis (*Arabidopsis thaliana*) mutants in four AG β-glucuronyltransferases (GlcAT14A, -B, -D, and -E) to understand the role of glucuronidation of AG. AG isolated from *glcat14* triple mutants had a strong reduction in glucuronidation. AG from a *glcat14a/b/d* triple mutant had lower calcium binding capacity in vitro than AG from wild-type plants. Some mutants had multiple developmental defects such as reduced trichome branching. *glcat14a/b/e* triple mutant plants had severely limited seedling growth and were sterile, and the propagation of calcium waves was perturbed in roots. Several of the developmental phenotypes were suppressed by increasing the calcium concentration in the growth medium. Our results show that AG glucuronidation is crucial for multiple developmental processes in plants and suggest that a function of AGPs might be to bind and release cell-surface apoplastic calcium.

## Abstract (precis)

Isolation and characterization of Arabidopsis arabinogalactan protein biosynthesis mutants suggest that this cell-surface proteoglycan can provide calcium for intracellular signaling pathways.

## INTRODUCTION

**Figure 1.**
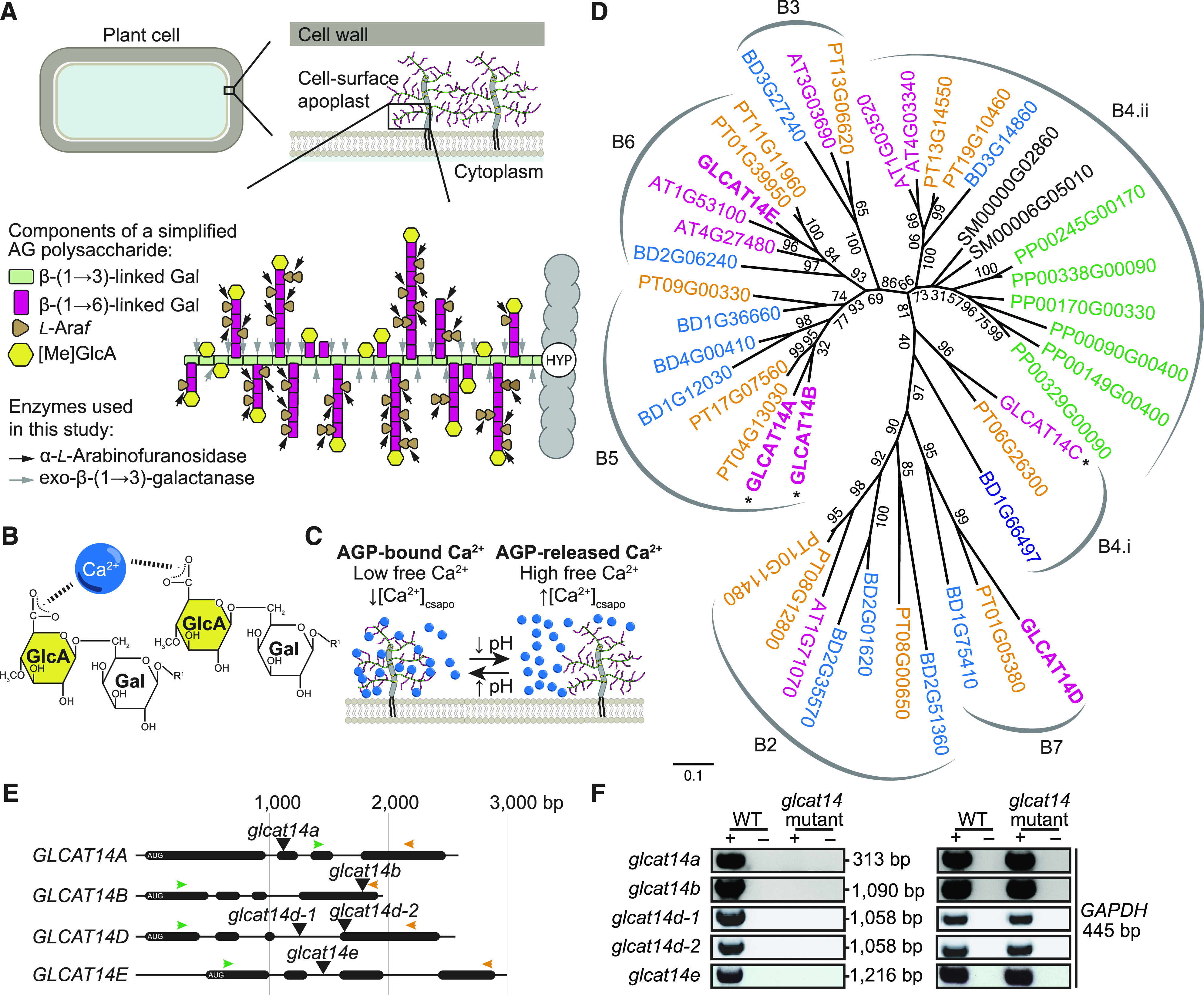
Strategy Followed for the Identification and Study of GlcATs. **(A)** Schematic representation of the localization of AGPs anchored on the extracellular side of the plasma membrane (the cell-surface apoplast) and a simplified putative model of the AG polysaccharide structure. An AG moiety is linked to a Hyp residue (HYP) from the protein core (gray) of AGPs. The AG β-(1→3)-galactan backbone (green rectangles) is substituted by β-(1→6)-galactan side chains (magenta rectangles), which is further decorated with α-l-arabinofuranose (l-Ara*f*) residues (brown triangles). [Me]GlcA residues (yellow hexagons) can decorate Gal on the β-(1→3)-galactan backbone or terminal Gal on the β-(1→6)-galactan side chains. The arrows indicate the hydrolysis sites of AG-specific enzymes, α-l-arabinofuranosidase (black) and exo-β-(1→3)-galactanase (gray), used in this study for the characterization of AG polysaccharides extracted from *glcat14* mutants. **(B)** Electrostatic interaction between AG polysaccharides and one Ca^2+^ ion via the C-6 carboxylate of two GlcA residues. The R1 group on Gal*p* can be linked to the β-(1→3)-galactan backbone or β-(1→6)-galactan side chains. **(C)** The AGP-Ca^2+^ capacitor model describes the reversible interaction between AGPs and Ca^2+^ at the cell surface. This interaction may be reversible in a pH-dependent manner, resulting in the increase or decrease in concentration of free Ca^2+^ at the cell-surface apoplast ([Ca^2+^]_csapo_; Lamport and Várnai, 2013). **(D)** The CAZy GT14 family is widespread across angiosperms. Three GT14s (denoted by asterisks) have a previously shown GlcAT activity. The protein sequences used in this phylogenetic tree were from Arabidopsis (AT; shown in magenta), *Physcomitrella patens* (PP; green), *Selaginella moellendorffii* (SM; black), *B. distachyon* (BD; blue), and *Populus trichocarpa* (PT; orange). Bootstrap replications = 1000. The clades were labeled as in previous reports (Ye et al., 2011; Pfeifer et al., 2020). The Arabidopsis protein sequences in boldface type were used in this study. **(E)** Representation of *GLCAT14A*, *GLCAT14B*, *GLCAT14D*, and *GLCAT14E* gene structures. The T-DNA insertion sites are indicated by black triangles. Arrows indicate the annealing positions of the forward primers (green) and reverse primers (orange) in RT-qPCR. **(F)** PCR products using leaf cDNA as a template to analyze the presence of the transcripts of *GLCAT14A*, *GLCAT14B*, *GLCAT14D*, and *GLCAT14E* in T-DNA insertion lines. Reverse transcriptase controls were used and labeled as positive (+) and negative (–) controls. The housekeeping gene *GAPDH* was used as a positive cDNA control for RT-qPCR.

Plant growth involves a wide number of processes that precisely control cell division, expansion, and differentiation. A family of molecules with a widely reported role in these fundamental processes are the arabinogalactan proteins (AGPs; Lalanne et al., 2004; Gillmor et al., 2005; Seifert and Roberts, 2007). AGPs are extracellular proteoglycans widespread across the plant kingdom (Ma et al., 2017), and they are found in all plant tissues (Knox et al., 1991) and cells (Pennell et al., 1991; Coimbra et al., 2009). AGPs are part of the Hyp-rich glycoprotein superfamily that includes extensins, Pro-rich proteins, and hybrid Hyp-rich glycoproteins, each of which has distinctive glycosylation motifs with distinctive carbohydrate moieties (Kieliszewski and Lamport, 1994; Fowler et al., 1999; Kieliszewski, 2001). The protein sequences of AGPs contain a secretion signal peptide at the N terminus, multiple arabinogalactan (AG) glycosylation motifs, and often a structural or enzymatic domain (Seifert and Roberts, 2007). The sequences of many proteins also direct the addition of a glycosylphosphatidylinositol (GPI) anchor at the C terminus. The GPI anchor attaches the proteins at the extracellular face of the plasma membrane (Youl et al., 1998; Sherrier et al., 1999; Borner et al., 2003), forming a narrow AGP-rich region between the cell membrane and the cell wall proper (Knox et al., 1989; Freshour et al., 1996), an apoplast region hereafter we call the cell-surface apoplast (Figure 1). AGP protein sequences are highly diverse, with at least 85 AGPs encoded in the Arabidopsis (*Arabidopsis thaliana*) genome (Showalter et al., 2010). Since additional GPI-anchored proteins may contain AG glycosylation motifs (Borner et al., 2002, 2003), the diversity of AGPs could be even greater. AGPs contain chemically similar carbohydrate moieties and are functionally redundant in biological processes (Ellis et al., 2010; Tan et al., 2012; Knoch et al., 2014), attributes that make AGPs challenging to study; consequently, little is known about their general molecular function (Tan et al., 2012).

**Figure:**
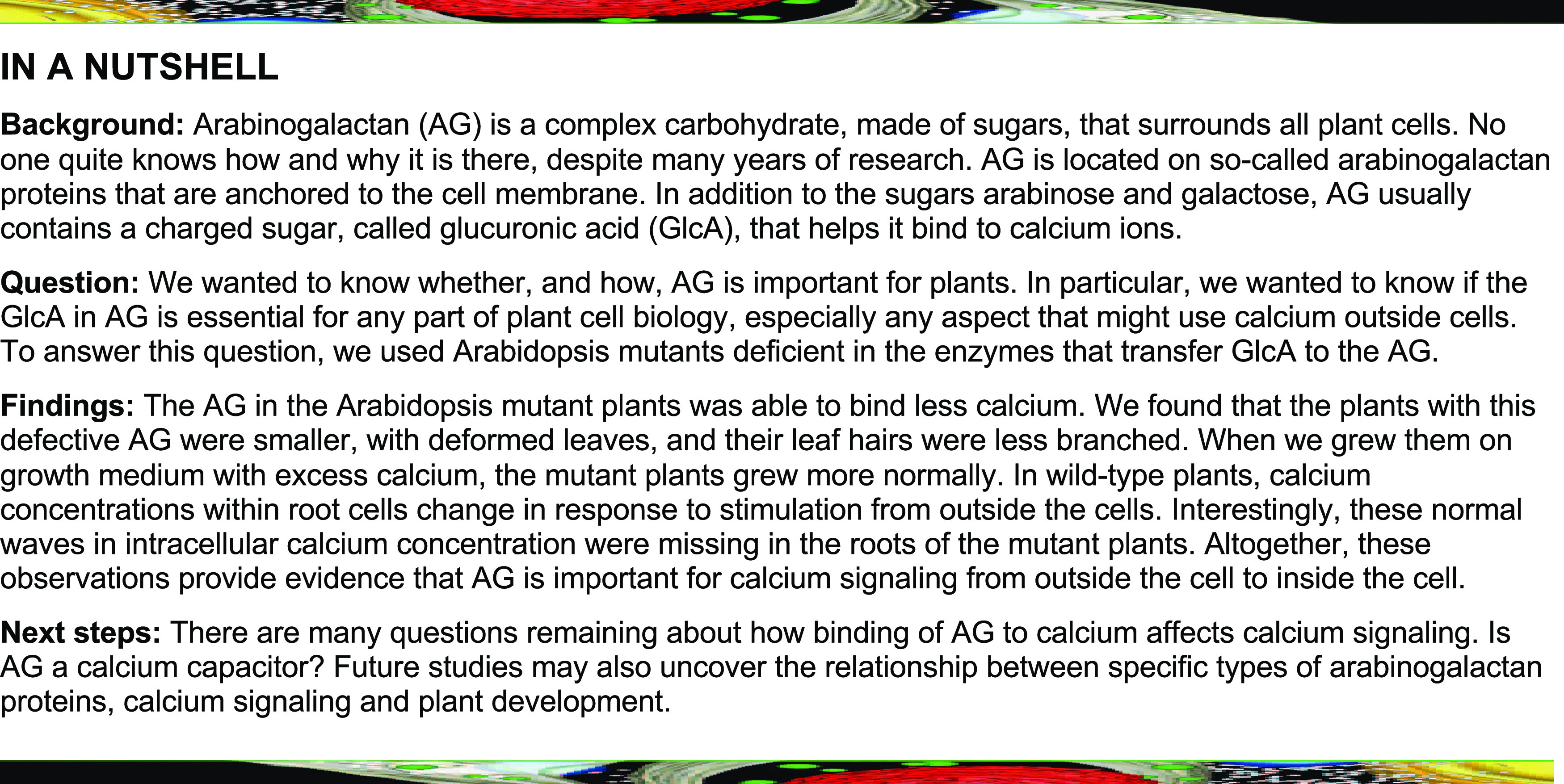


The carbohydrate moieties of AGPs are AG polysaccharides. AGs are *O*-linked to one or more Hyp residues of the protein core of AGPs (Figure 1A; Du et al., 1994). These type II AGs are composed of a distinctive β-(1→3)-galactan backbone that is further substituted by β-(1→6)-galactan side chains (Anderson et al., 1977). The side chains are always highly modified with α-l-arabinofuranose (Tsumuraya et al., 1984; Tryfona et al., 2010), and AG is usually also decorated with β-glucuronic acid (GlcA) that can be methylated (4-*O*-Me-Glc*p*A; MeGlcA); here, both forms are referred to as [Me]GlcA (Haque et al., 2005). In addition, AG may contain further minor sugars such as l-arabinopyranose, l-fucose, l-rhamnose, and xylopyranose (Tsumuraya et al., 1984; Ponder and Richards, 1997; Tan et al., 2004; Tryfona et al., 2010, 2014).

[Me]GlcA residues are mainly found terminating the β-(1→6)-galactan side chains of AG, although this glucuronidation is also found on the β-(1→3)-galactan backbone (Figure 1A; Tan et al., 2004; Tryfona et al., 2012). This key location at the surface of the AG structure, and their near ubiquitous presence (Tsumuraya et al., 1984; Lamport and Várnai, 2013), suggest that glucuronidation may confer functionally relevant properties on AGPs. First, [Me]GlcA residues may give AGPs the ability to bind reversibly to Ca^2+^ ions (Figures 1B and 1C; Lamport and Várnai, 2013). Second, GlcA may terminate the elongation of the β-(1→6)-galactan side chains during biosynthesis (Knoch et al., 2013). Third, GlcA forms a bridging residue between pectin and AGPs, as shown in the APAP1 molecule, and so might be important in the assembly of complex proteoglycans (Tan et al., 2013). Fourth, MeGlcA was recently identified in *Torenia fournieri* to be an essential moiety of the disaccharide 4-*O*-Me-GlcA-β-(1→6)-Gal, called AMOR, which is required for the pollen tube’s competency to perceive LURE attractant peptides in vitro (Mizukami et al., 2016). While AMOR highlights a biological role of MeGlcA on AGs for plant reproduction, its function has not yet been identified in vegetative tissues. Thus, the availability of mutants with reduced amounts of [Me]GlcA on AG polysaccharides could lead to the discovery of more general functional aspects of AGPs in plants. Various enzymes involved in AG polysaccharide biosynthesis have been reported, but many remain unidentified. The AG-specific glycosyltransferases (GTs) characterized include eight galactosyltransferases (GalTs; GALT2 to -6 and HPGT1 to -3) that transfer galactose onto Hyp (Basu et al., 2013, 2015; Ogawa-Ohnishi and Matsubayashi, 2015), two GalTs (GALT31A and GALT29A) involved in the synthesis of β-(1→6)-galactan side chains (Geshi et al., 2013; Dilokpimol et al., 2014), two backbone β-(1→3)-Gal transferases (UPEX1 and GhGalT1; Qin et al., 2017; Suzuki et al., 2017), and two l-fucosyltransferases (FUT4 and FUT6; Liang et al., 2013; Tryfona et al., 2014). Recently, the terminal GlcA was described to be methylated by two 4-*O*-methyltransferases (AGM1 and AGM2) of the DUF579 family (Temple et al., 2019). Together, these enzymes are likely responsible for the glycosylation of a large number of proteins encoded in the Arabidopsis genome (Borner et al., 2003; Showalter et al., 2010). Given the high redundancy of AGP backbones and the AG biosynthetic enzymes, double and triple mutants have been required to observe growth phenotypes such as increased salt sensitivity (Liang et al., 2013; Tryfona et al., 2014), reduced inflorescence growth (Ogawa-Ohnishi and Matsubayashi, 2015), and defective pollen development (Coimbra et al., 2009). Any general biological role and molecular function of AGPs remain unknown (Tan et al., 2012).

Three of the 11-member CAZy GT family 14 enzymes in Arabidopsis (GLCAT14A to -C) have been shown in vitro to possess β-(1→6)-GlcA transferase (GlcAT) activity on both the β-(1→3)-galactan backbone and β-(1→6)-galactan side chains, but with a distinct substrate preference (Knoch et al., 2013; Dilokpimol and Geshi, 2014). The *glcat14a* mutant had reduced glucuronidation of AGs, confirming the GlcAT activity of GLCAT14A in vivo. GLCAT14A and -B were described to act preferentially on the β-(1→6)-galactan side chains, whereas GLCAT14C prefers the β-(1→3)-galactan backbone (Dilokpimol and Geshi, 2014). Null mutants in *GLCAT14A* showed only mild growth defects in etiolated seedlings (Knoch et al., 2013). The substantial residual glucuronidation of AGs extracted from the *glcat14a* mutants suggested that additional GlcATs may be redundant to GLCAT14A in Arabidopsis. Alternatively, the GlcATs may glycosylate AGPs with different types of backbones or they may glycosylate different positions of the AG glycan. Therefore, the specificity of the enzymes for certain AGPs or AG structures, and the general importance of glucuronidation of AG polysaccharides, remain to be clarified.

Here, our aim was to study the biological role of glucuronidation of AG polysaccharides. The property of [Me]GlcA having a pH-dependent AGP interaction with Ca^2+^ ions in vitro (Figures 1B and 1C) gave rise to the AGP-Ca^2+^ capacitor hypothesis (Lamport and Várnai, 2013). This hypothesis proposes that glucuronidated AGPs interact with Ca^2+^ and can potentially release Ca^2+^, contributing to cellular Ca^2+^ oscillations and plant growth (Lamport and Várnai, 2013). Ca^2+^ has at least two roles in the organism: structural, such as the millimolar levels of Ca^2+^ that are found in the cell wall bound to gelled pectin (Demarty et al., 1984; Willats et al., 2001); and as a second messenger due to nanomolar changes in the free Ca^2+^ concentration that mediate signaling in the cytosol and organelles (Kudla et al., 2018). As AGPs are abundant in the cell-surface apoplast (Borner et al., 2003), any release of Ca^2+^ bound to the AG is immediately available to the plant cell. Buffering of extracellular Ca^2+^ by AGPs could contribute to the maintenance of low cytosolic free Ca^2+^ ([Ca^2+^]_cyt_), which is kept in the 100 to 200 nM range to avoid cytotoxicity by phosphate precipitation. However, Ca^2+^ binding by AGPs might also contribute to the regulation of signaling, because the period and shape of oscillations of [Ca^2+^]_cyt_ that encode information in signaling networks are sensitive to cell-surface apoplastic free [Ca^2+^] ([Ca^2+^]_csapo_) due to the flux of Ca^2+^ into the cytosol across the plasma membrane (McAinsh et al., 1995). If not properly regulated, this Ca^2+^ can inappropriately affect plant performance through effects on signaling and homeostasis, and this regulation is sensitive to the soil Ca^2+^ concentration (Conn et al., 2011). Thus, AGPs might serve as a reservoir of Ca^2+^ for processes such as Ca^2+^ signaling and cell expansion (Lamport et al., 2018a), with the possibility that release of Ca^2+^ from cell-surface AGPs will trigger changes in [Ca^2+^]_cyt_ dynamics (McAinsh et al., 1995). To test this hypothesis, we identified Arabidopsis GlcAT mutants from the GT14 family with altered AG glucuronidation. We found that the substantially reduced content of [Me]GlcA on AGs in *glcat14* triple mutants led to several deficiencies in plant development and in the spatiotemporal propagation of Ca^2+^ waves. By growing mutants in increasing concentrations of Ca^2+^, the developmental phenotypes were suppressed, suggesting that the developmental phenotypes arise from deficiencies in Ca^2+^ binding by poorly glucuronidated AGPs and, consequently, in intracellular Ca^2+^ signaling.

## RESULTS

### Identification of Candidate Arabidopsis AG GlcATs and GlcAT Mutants

To select the putative Arabidopsis AG GlcATs for mutant studies, a phylogenetic tree of the CAZy GT14 family was built (Figure 1D; Supplemental File). For improved robustness of this phylogeny, in addition to the 11 Arabidopsis GT14 enzymes, homologous sequences from other plant species were included. We identified seven clades in the phylogenetic tree, each of which consists of at least one Arabidopsis, *Brachypodium distachyon*, and poplar (*Populus trichocarpa*) protein. These seven clades have both eudicot and monocot angiosperm GT14 members, suggesting a possible conserved divergence of function. The phylogeny also highlighted possible genetic redundancy of some of the enzymes of the GT14 family in Arabidopsis, including GLCAT14A and GLCAT14B.

A weak *glcat14a* mutant growth phenotype has been observed, and a small reduction in [Me]GlcA was noted in the AG extracted from the roots (Knoch et al., 2013). To select further candidate Arabidopsis GlcATs that are expressed in rosette leaves and roots, which are tissues amenable to biochemical analysis of AGs (Tryfona et al., 2012, 2014; Knoch et al., 2013), gene expression levels of the members of the GT14 family were compared (Supplemental Figure 1; Waese et al., 2017). The top two expressed genes in leaves and roots, which we named *GLCAT14D* and *GLCAT14E*, were selected for study. *AT1G71070* expression is high in roots and other tissues, but no T-DNA insertion lines were available for this gene. We also selected *GLCAT14B* because its encoded protein is 73% identical to GLCAT14A (Figure 1D; Supplemental Figure 2) and its GlcAT activity has been demonstrated in vitro (Dilokpimol and Geshi, 2014). No activity data are available for GLCAT14D and GLCAT14E, but GLCAT14B, GLCAT14D, and GLCAT14E have been localized to the Golgi apparatus, where AG glucuronidation occurs (Lao et al., 2014).

To study the function of the selected putative GlcATs in Arabidopsis, homozygous T-DNA insertion null mutants were identified for *GLCAT14B* (one line), *GLCAT14D* (two lines), and *GLCAT14E* (one line; Figures 1E and 1F). For *GLCAT14A*, we used the previously reported null mutant (Knoch et al., 2013). Because only one line was available, genetic complementation of the *glcat14b* and *glcat14e* mutants is presented in the following sections.

### Both GLCAT14A and GLCAT14B Contribute to Glucuronidation of AG in Vivo

**Figure 2.**
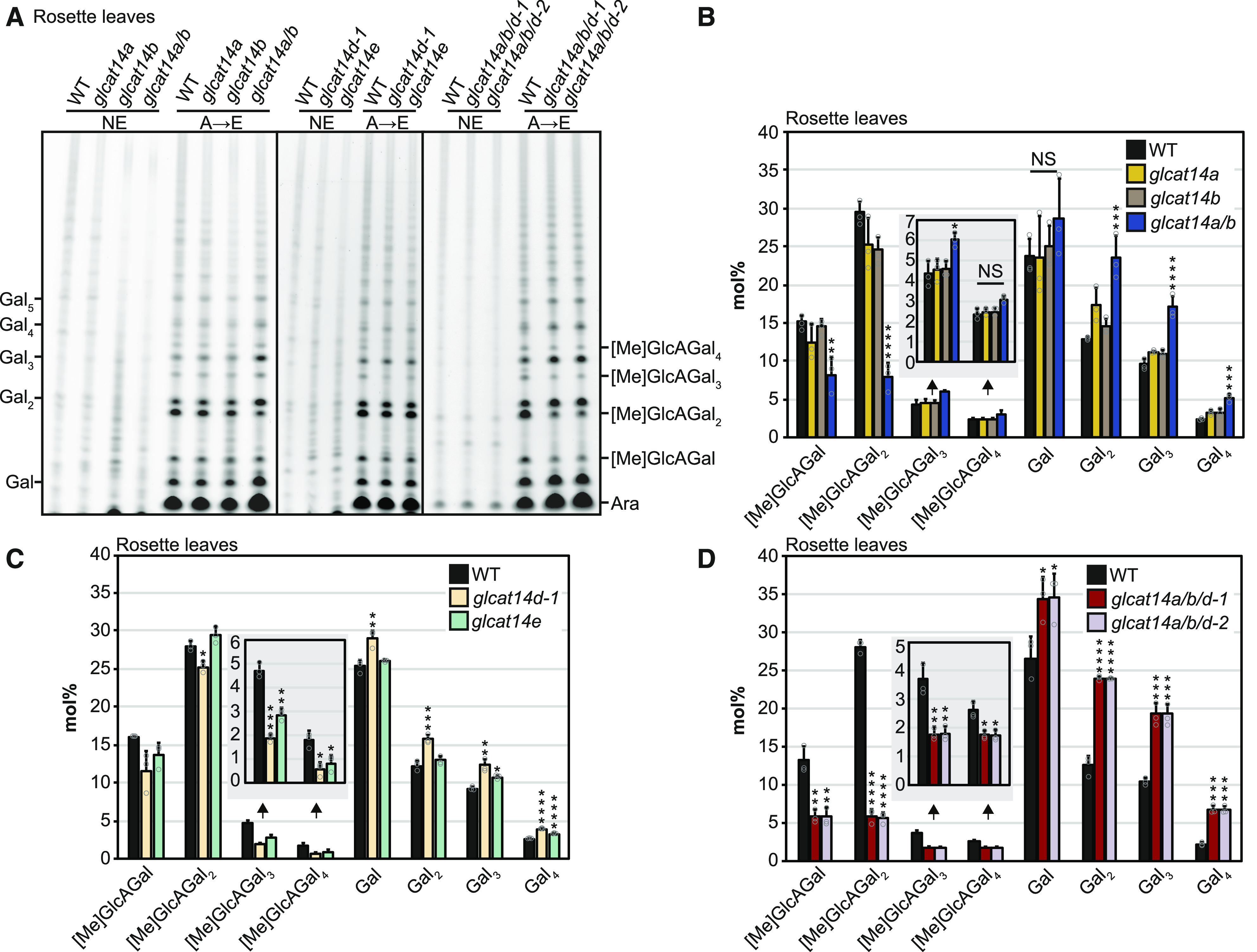
[Me]GlcA-Containing Oligosaccharides Are Reduced in Digests of Rosette Leaf AGs from *glcat14* Mutants. **(A)** PACE analysis of rosette leaf AG extracts from the wild type, *glcat14a*, *glcat14b*, *glcat14a/b*, *glcat14d-1*, *glcat14e*, *glcat14a/b/d-1*, and *glcat14a/b/d-2*. AG oligosaccharides were released by the sequential hydrolysis with AG-specific α-l-arabinofuranosidase (A) followed by exo-β-(1→3)-galactanase (E). NE, no enzyme control. **(B)** to **(D)** The intensities of oligosaccharides from PACE **(A)** were quantified to determine the abundance expressed in mol% of galactose, β-(1→6)-galactobiose, β-(1→6)-galactotriose, β-(1→6)-galactotetraose, and oligosaccharides substituted by [Me]GlcA of the same galactan length. Insets show the abundance of glucuronidated β-(1→6)-galactotriose and β-(1→6)-galactotetraose. The data are from wild-type and mutant plants grown alongside each other. Values are means ± sd from three biological replicates. Asterisks indicate significant differences between mutants and the wild type defined by one-way ANOVA followed by Tukey’s multiple comparison test: *, P < 0.05; **, P < 0.01; ***, P < 0.001; and ****, P < 0.0001. NS, not significant.

**Figure 3.**
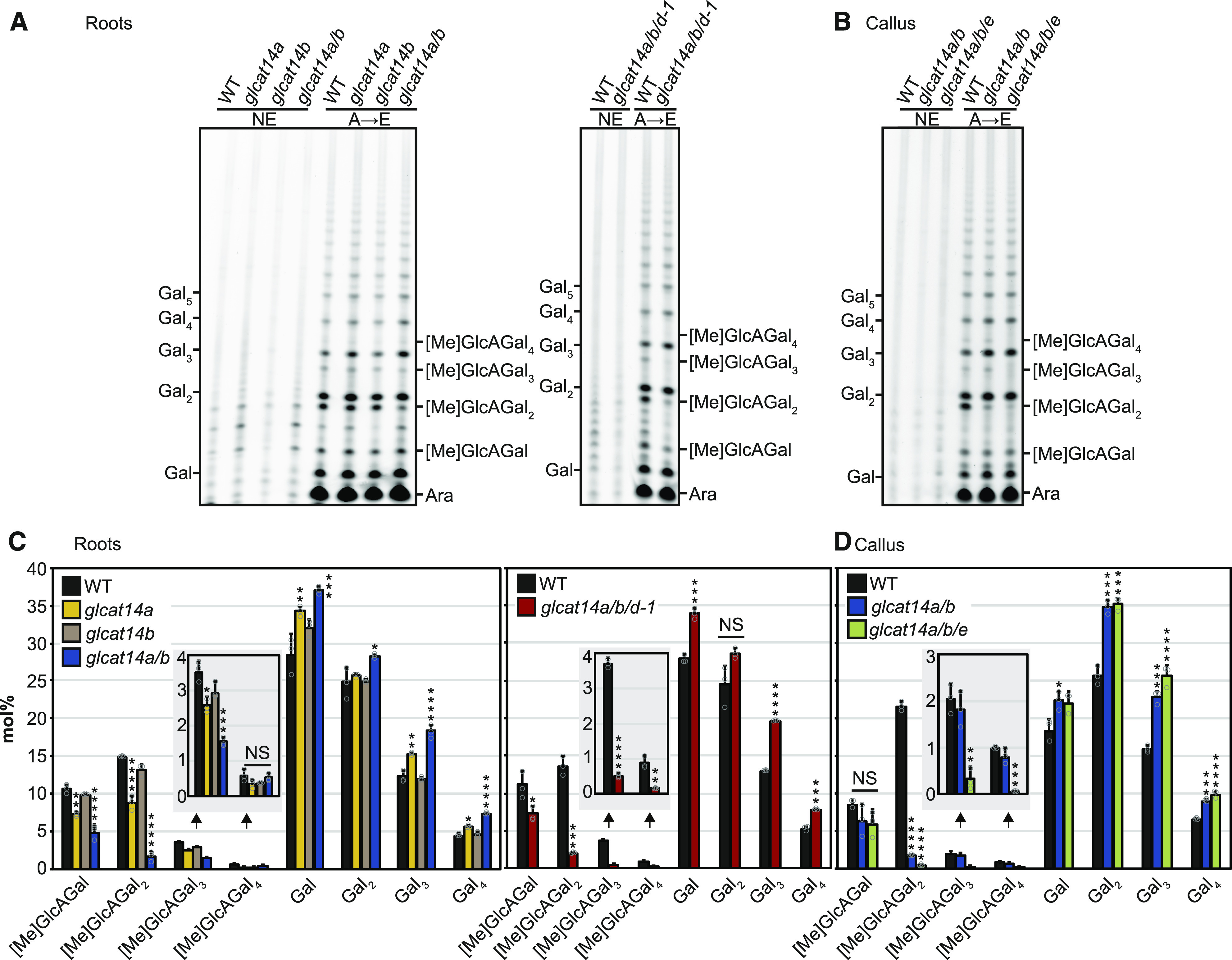
[Me]GlcA-Containing Oligosaccharides Are Reduced in Digests of Root and Callus AGs from *glcat14* Mutants. **(A)** PACE analysis of root AG digests from the wild type, *glcat14a*, *glcat14b*, *glcat14a/b*, and *glcat14a/b/d-1*. **(B)** PACE analysis of callus AG digests from the wild type, *glcat14a/b*, and *glcat14a/b/e*. **(A)** and **(B)** were analyzed as in Figure 2A. **(C)** and **(D)** The intensities of oligosaccharides from PACE (**[A]** and **[B]**) were quantified to determine the abundance expressed in mol% of galactose, β-(1→6)-galactobiose, β-(1→6)-galactotriose, β-(1→6)-galactotetraose, and oligosaccharides substituted by [Me]GlcA of the same galactan length. Insets show the abundance of glucuronidated β-(1→6)-galactotriose and β-(1→6)-galactotetraose. The data are from wild-type and mutant plants grown alongside each other. Values are means ± sd from three biological replicates. Asterisks indicate significant differences between mutants and the wild type defined by one-way ANOVA followed by Tukey’s multiple comparison test or Student’s *t* test for two-sample comparisons: *, P < 0.05; **, P < 0.01; ***, P < 0.001; and ****, P < 0.0001. NS, not significant.

To explore whether GLCAT14B functions as a GlcAT and to investigate any redundancy with GLCAT14A, rosette leaf and root AGPs were extracted from *glcat14a*, *glcat14b*, and the *glcat14a/b* double mutant. The [Me]GlcA frequency on AG side chains was measured by polysaccharide analysis using carbohydrate gel electrophoresis (PACE), using enzymatic hydrolysis to release short β-(1→6)-galactooligosaccharides that may have terminal [Me]GlcA (Figures 1A, 2, and 3). The products were identified by comigration with previously reported oligosaccharides (Tryfona et al., 2012; Knoch et al., 2013; Shimoda et al., 2014). The intensity of the bands corresponding to [Me]GlcAGal_1-4_ and Gal_1-4_ was quantified. To estimate changes in the proportion of glucuronidated and nonglucuronidated AG species between the wild type and *glcat14* mutants, the percentage of each oligosaccharide abundance was determined (Figures 2B to 2D, 3C, and 3D). Any glucuronidated oligosaccharides of higher degree of polymerization (DP) were not quantified. In root AGPs from *glcat14a*, we confirmed the previously reported reduction of [Me]GlcAGal and [Me]GlcAGal_2_ (Knoch et al., 2013). In leaves, the activity of GLCAT14A and GLCAT14B was evident in the *glcat14a/b* double mutant, in which the abundance of the [Me]GlcAGal and [Me]GlcAGal_2_ oligosaccharides was reduced to one-half and one-fourth of wild-type levels, respectively (Figure 2B). These results indicate that GLCAT14A is active in leaves in addition to the previously reported activity in roots (Knoch et al., 2013). In AGPs from *glcat14a/b* roots, the abundance of [Me]GlcAGal_1-3_ decreased below the levels present in *glcat14a* mutants (Figure 3C), indicating that the GlcAT activity of GLCAT14B is partly redundant to GLCAT14A in vivo, as suggested by Dilokpimol and Geshi (2014). The consistent reduction in [Me]GlcAGal and [Me]GlcAGal_2_ suggests that GLCAT14A and GLCAT14B preferentially glucuronidate the β-(1→3)-galactan backbone and the short single residue β-(1→6)-linked galactan side chains.

### GLCAT14D and GLCAT14E Are Important for AG Glucuronidation

To investigate whether GLCAT14D and GLCAT14E are important for AG glucuronidation in vivo, leaf AGPs from *glcat14d-1* and *glcat14e* mutants were analyzed by PACE (Figures 2A and 2C). The abundance of [Me]GlcAGal_2_ was significantly lower in AGP hydrolysates from *glcat14d-1* compared with the wild type. Moreover, hydrolysis of both *glcat14d-1* and *glcat14e* AGPs showed a large reduction of [Me]GlcAGal_3-4_. This suggests that GLCAT14D and GLCAT14E are GlcATs preferentially involved in the glucuronidation of longer AG side chains.

### AGP Glucuronidation Is Strongly Reduced in *glcat14a/b/d* and *glcat14a/b/e* Triple Mutants

To explore whether mutations of either of GLCAT14D or GLCAT14E would further reduce the amount of [Me]GlcA in *glcat14a/b* double mutants, the triple mutants *glcat14a/b/d* and *glcat14a/b/e* were generated. For *glcat14a/b/d*, two triple mutant lines were generated using two independent null alleles of *glcat14d* (Figures 1E and 1F). Leaf AG extracts from *glcat14a/b/d-1* and *glcat14a/b/d-2* were enzymatically hydrolyzed and analyzed by PACE (Figure 2). AGs in both the *glcat14a/b/d-1* and *glcat14a/b/d-2* triple mutants had substantially reduced glucuronidation of Gal_1-4_ in leaves (Figures 2A and 2D). Similarly, root AGPs from *glcat14a/b/d-1* triple mutants showed lower amounts of glucuronidation than the single and *glcat14a/b* double mutants, being reduced in all four oligosaccharides (Figures 3A and 3C).

The growth of the *glcat14a/b/e* triple mutant was poor (discussed further below), hindering the analysis of AG from leaves or roots. Previous studies support the use of cell cultures such as callus as a good source of AGs (Serpe and Nothnagel, 1994; Sherrier et al., 1999; Lamport et al., 2006), and gene expression data indicate that *GLCAT14A*, *GLCAT14B*, and *GLCAT14E* are expressed in callus (Supplemental Figure 1). Therefore, a callus liquid culture was generated from *glcat14a/b/e* seedling roots. We also generated callus from the *glcat14a/b* double mutant and used it as a reference to determine the contribution of GLCAT14E in AG glucuronidation. Callus AG extracts from the wild type, *glcat14a/b*, and *glcat14a/b/e* were analyzed by PACE (Figure 3B). Glucuronidation of AGs from *glcat14a/b/e* mutants was less than that of AGs from *glcat14a/b* double mutants, particularly in the glucuronidated oligosaccharides of Gal_2-4_ (Figure 3D).

**Figure 4.**
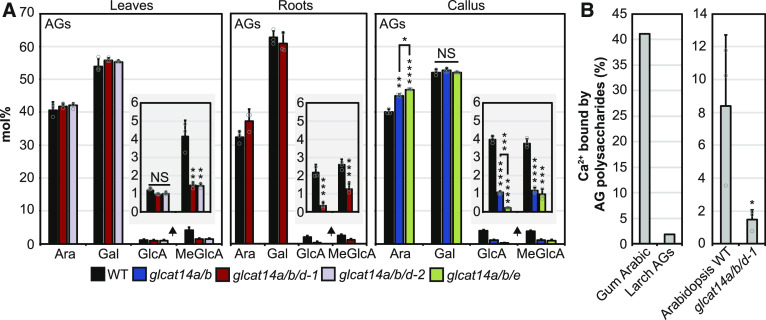
The Reduction of [Me]GlcA in AG Polysaccharides Significantly Reduces the Ca^2+^ Binding Capacity in Vitro. **(A)** HPAEC-PAD monosaccharide composition of AGs extracted from rosette leaves, mature roots, and callus from the wild type, *glcat14a/b*, *glcat14a/b/d-1*, *glcat14a/b/d-2*, and *glcat14a/b/e*. Monosaccharides and β-(1→6)-galactobiose were enzymatically released by α-l-arabinofuranosidase, exo-β-(1→3)-galactanase, endo-β-(1→3)-galactanase, endo-β-(1→6)-galactanase, and GUS. Insets show the abundance of GlcA and MeGlcA. For estimating total Gal, the amount of β-(1→6)-galactobiose was summed up as two Gal molecules. Values are means ± sd from three biological replicates. Asterisks indicate significant differences between mutants and the wild type defined by one-way ANOVA followed by Tukey’s multiple comparison test: **, P < 0.01; ***, P < 0.001; and ****, P < 0.0001. NS, not significant. **(B)** Percentage of Ca^2+^ bound to 2.5 mg of AGs from gum arabic, larch AGs, and Arabidopsis wild-type and *glcat14a/b/d-1* rosette leaf AGs resuspended in 450 μL of 10 mM ammonium acetate, pH 5.5, containing 2 mM CaCl_2_. The Ca^2+^ bound to AGs was determined by ICP-MS analysis. The graphs represent the Ca^2+^ binding capacity of one replicate of gum arabic and larch AGs (left) and the mean ± sd of three biological replicates of Arabidopsis AGs (right). The wild-type and mutant samples shown here were extracted from plants grown alongside each other. The asterisk above the bar indicates a significant statistical difference defined by Student’s *t* test: *, P < 0.1.

To investigate further the reduction of glucuronidation of AG in the *glcat14* mutants, an extensive enzymatic hydrolysis followed by high-performance anion-exchange chromatography with pulsed amperometric detection (HPAEC-PAD) was used to determine the amounts of Gal, Ara, GlcA, and MeGlcA in AG polysaccharides. Enzymes were used in preference to acid hydrolysis, which does not hydrolyze effectively the glycosidic linkage between [Me]GlcA and Gal, leading to underestimation of [Me]GlcA (Gloaguen et al., 1997; Ogawa et al., 1998). Thus, AG polysaccharides were hydrolyzed with α-l-arabinofuranosidase, exo-β-(1→3)-galactanase, endo-β-(1→3)-galactanase, endo-β-(1→6)-galactanase, and GUS (Konishi et al., 2008; Kotake et al., 2009; Takata et al., 2010; Yoshimi et al., 2017). The monosaccharides in AGs from *glcat14a/b/d-1* and *glcat14a/b/d-2* leaves and roots, as well as from *glcat14a/b* and *glcat14a/b/e* callus, are shown in Figure 4. The reduction of glucuronidation detected by PACE was confirmed, and this analysis also showed that both GlcA and MeGlcA were reduced in roots and callus. MeGlcA, but not GlcA, was reduced in leaf AG, suggesting that the AG polysaccharide structures glucuronidated by these enzymes are preferentially methylated. The estimated overall reduction of glucuronidation in *glcat14* mutants compared with wild-type plants was as follows: for leaves, *glcat14a/b/d-1* 55% and *glcat14a/b/d-2* 54%; for roots, *glcat14a/b/d-1* 66%; for callus, *glcat14a/b* 71% and *glcat14a/b/e* 85%. The proportions of Ara and Gal showed minor or no changes in the *glcat14* triple mutant AGs (Figure 4A), which was confirmed in acid-hydrolyzed AG extracts studied by HPAEC-PAD (Supplemental Figure 3A). To explore any general changes in cell wall polysaccharides, we analyzed trifluoroacetic acid (TFA)-hydrolyzed alcohol-insoluble residue from rosette leaves and callus of *glcat14* double and triple mutants (Supplemental Figure 3B). We detected minor changes mainly in *glcat14* triple mutants, but the overall profile of cell wall sugar composition was similar to wild-type alcohol-insoluble residue.

### Calcium Binding to AG in Vitro Is Reduced in AG Glucuronidation Mutants

An interaction between AGPs and Ca^2+^ is suggested to occur through the AG [Me]GlcA in a pH-dependent manner (Figure 1; Lamport and Várnai, 2013). Thus, the reduced glucuronidation of AGs in the *glcat14* triple mutants should have consequences for any AGP-Ca^2+^ interaction, and therefore an in vitro Ca^2+^ binding assay was performed. Since homogalacturonan is known to bind Ca^2+^ (Willats et al., 2001), this pectin was thoroughly removed from the Arabidopsis AG extracts, so that the content of GalA was below 2 mol% measured by HPAEC-PAD after acid hydrolysis. The AGP gum arabic was used as a positive control because it contains high amounts of [Me]GlcA (Lluveras-Tenorio et al., 2012). On the other hand, larch (*Larix* spp.) AG was used as a negative control because it has negligible glucuronidation (Trofimova et al., 2012; Lamport and Várnai, 2013). The Ca^2+^ binding capacity of Arabidopsis leaf AGs from the wild type and *glcat14a/b/d-1* triple mutants was determined with inductively coupled plasma mass spectrometry (ICP-MS; Figure 4B). The AGs from *glcat14a/b/d-1* mutants bound ∼80% less Ca^2+^ compared with wild-type plants. This supports the hypothesis that glucuronidation is required for Ca^2+^ to bind to AGPs.

### The Reduction in AG Glucuronidation Causes Pleiotropic Growth Defects

**Figure 5.**
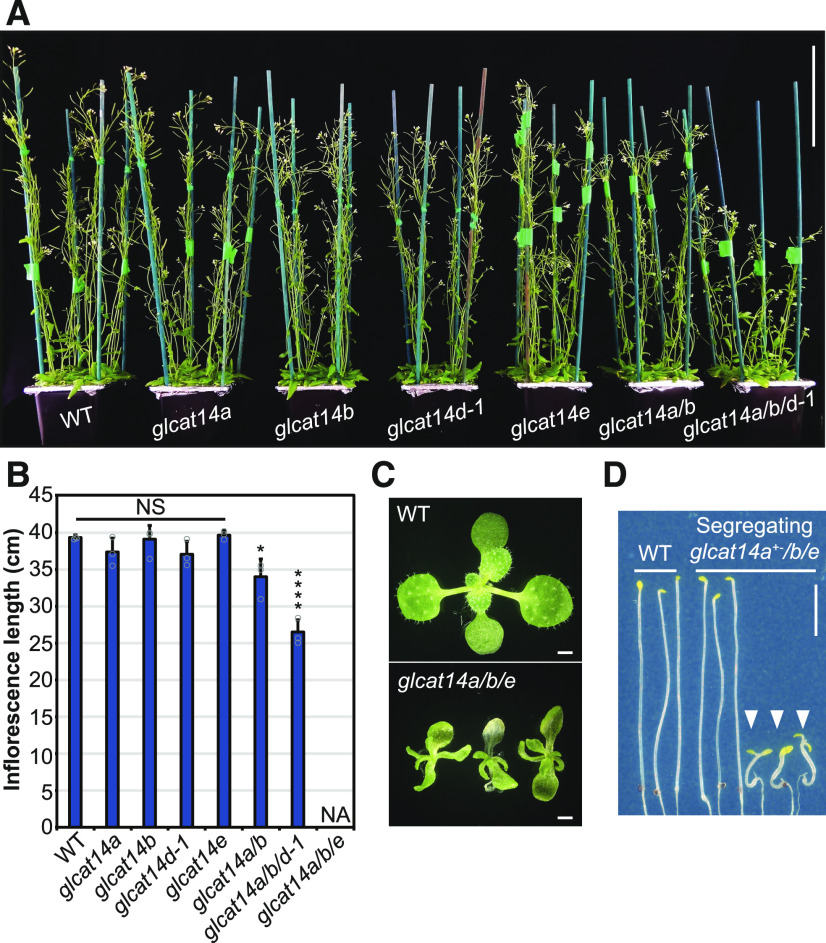
Impaired Growth of *glcat14a/b/d* and *glcat14a/b/e* Triple Mutants. **(A)** Five-week-old plants from the wild type and *glcat14* mutants grown in hydroponic solution. Bar = 10 cm. **(B)** Inflorescence stem lengths from wild-type and *glcat14* mutant plants grown in hydroponic solution. NA, data not available because the plants did not grow stems. Data represent means ± sd of three biological replicates. *n* = 16 per line per replicate. Asterisks indicate significant differences between mutants and the wild type defined by one-way ANOVA followed by Tukey’s multiple comparison test: *, P < 0.05 and ****, P < 0.0001. NS, not significant. **(C)** Fifteen-day-old seedlings from the wild type and *glcat14a/b/e* mutants grown on basal MS medium. Bars = 1 mm. **(D)** Nine-day-old dark-grown hypocotyls from the wild type and segregating *glcat14a*^*+−*^*/b/e* mutants grown on basal MS medium. White arrowheads indicate homozygous *glcat14a/b/e* mutant seedlings. Note the deetiolated phenotype on *glcat14a/b/e*. Bar = 0.5 cm.

To explore the biological importance of glucuronidation of AGs, the growth phenotypes of the mutants were studied. First, the inflorescence stem length was measured in 5-week-old plants of single mutants (*glcat14a*, *glcat14b*, *glcat14d-1*, and *glcat14e*), the *glcat14a/b* double mutant, and the *glcat14a/b/d-1* triple mutant (Figures 5A and 5B). No evident growth phenotype in inflorescence stem lengths was identified in *glcat14a*, *glcat14b*, *glcat14d-1*, and *glcat14e* single mutants under standard growth conditions. In contrast, the *glcat14a/b* double and *glcat14a/b/d-1* triple mutants were ∼10 and 30% shorter than wild-type plants, respectively.

The growth of *glcat14a/b/e* mutants was severely deficient, limiting the generation of progeny (Figure 5C). Therefore, the heterozygous *glcat14a*^*+−*^*/b/e* line was used to obtain *glcat14a/b/e* triple homozygous mutants by segregation. *glcat14a*^*+−*^*/b/e* segregated in a Mendelian manner, with one-fourth of the progeny being *glcat14a/b/e* triple homozygous. The growth of *glcat14a/b/e* triple mutants was slower than that of wild-type plants, with characteristic slender and curved leaves (Figure 5C). Seedlings from *glcat14a/b/e* could be grown in basal Murashige and Skoog (MS) medium, but growth ceased after ∼15 d.

Previously, it was reported that dark-grown seedlings had longer hypocotyls and roots in null mutants of *GLCAT14A* than wild-type seedlings (Knoch et al., 2013). Gene expression patterns suggested that *GLCAT14B*, *GLCAT14D*, and *GLCAT14E* are also expressed in dark-grown seedlings (Supplemental Figure 1). Thus, we measured the hypocotyl length of dark-grown seedlings from *glcat14* double and triple mutants (*glcat14a/b*, *glcat14a/b/d*, and *glcat14a/b/e*). No differences in hypocotyl length were found between the wild type, *glcat14a/b*, and *glcat14a/b/d-1* (discussed below), whereas *glcat14a/b/e* hypocotyls were remarkably shorter than wild-type and segregating *glcat14a*^*+−*^*/b/e* hypocotyls (Figure 5D). The dark-grown seedlings of *glcat14a/b/e* lacked the typical etiolated phenotype, were crooked, and lacked an apical hook. The short hypocotyl, lack of apical hook, and open cotyledons of *glcat14a/b/e* are reminiscent of a mild deetiolated phenotype (Chory et al., 1989).

**Figure 6.**
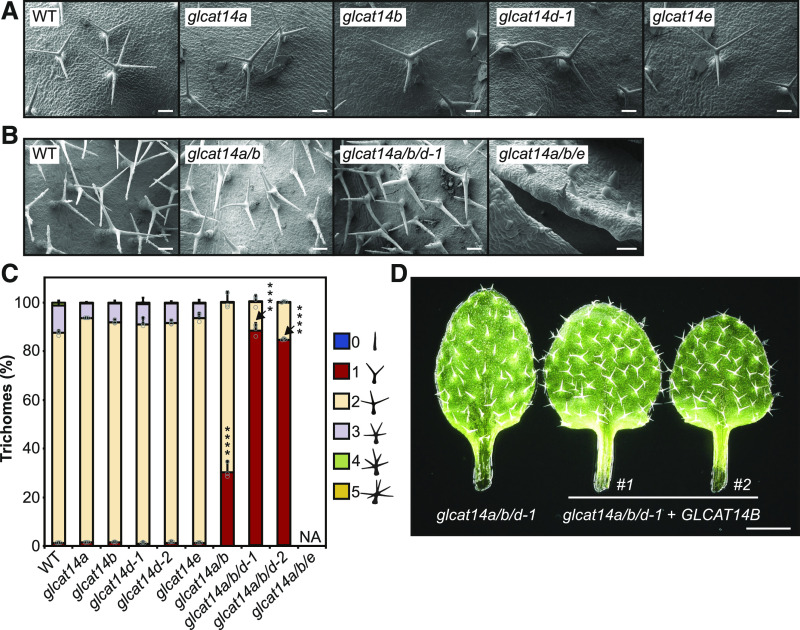
Trichomes of *glcat14a/b* and *glcat14a/b/d* Mutants Have Reduced Numbers of Branches. **(A)** and **(B)** CryoSEM micrographs of trichomes of the wild type, *glcat14a*, *glcat14b*, *glcat14d-1*, and *glcat14e* **(A)** and the wild type, *glcat14a/b*, *glcat14a/b/d*, and *glcat14a/b/e* **(B)** third true leaves from plants grown on basal MS medium. Bars = 100 μm. **(C)** Quantification of trichome branching points from third true leaves from the wild type and *glcat14* mutants. The percentage of trichomes by number of branching points was calculated. NA, data not available as trichomes did not branch. The graph represents means ± sd of three biological replicates. *n* = 10 leaves per line per replicate. Data points are shown for trichomes with one and two branching points. Asterisks indicate significant differences between mutants and the wild type defined by one-way ANOVA followed by Tukey’s multiple comparison test: ****, P < 0.0001. **(D)** Genetic complementation of *glcat14a/b/d-1*. Third true leaves of *GLCAT14B*-complemented lines #1 and #2 show normal trichome branching when compared with *glcat14a/b/d-1*. Bar = 1 mm.

Preliminary observations suggested that trichomes of some mutant plants had reduced branching. To explore the trichome phenotype, cryo-scanning electron microscopy (cryoSEM) images were taken of trichomes from single mutants (Figure 6A), the *glcat14a/b* double mutant, and the *glcat14a/b/d-1* and *glcat14a/b/e* triple mutants (Figure 6B), and the branching was quantified (Figure 6C). Single mutants had trichomes similar to the wild type, with two branching points. In contrast, *glcat14a/b* and *glcat14a/b/d* mutants had an increased proportion of trichomes with one branching point. The *glcat14a/b/d* mutants had a larger number of trichomes with one branching point, whereas in *glcat14a/b/e* mutants, trichome development was severely affected and branching was not evident. This suggests that the activity of GLCAT14A, -B, -D, and -E is required for trichome branching or development in Arabidopsis.

To confirm the importance of GLCAT14B, where only one mutant allele was available, genetic complementation was performed for *glcat14a/b/d*. For this, we used the suppression of the *glcat14a/b/d* trichome phenotype as one that could be easily scored. Analysis of two independent *GLCAT14B*_*pro*_:*GLCAT14B-GFP glcat14a/b/d-1* transgenic lines showed that the wild-type copy of the gene could fully complement the trichome-branching phenotype, restoring wild-type behavior (Figure 6D). Thus, the genetic complementation of *GLCAT14B* confirmed that the *glcat14a/b/d* mutant phenotypes arise from mutagenesis of this gene.

### Calcium Suppresses Growth and Developmental Phenotypes of AG Glucuronidation Mutants

**Figure 7.**
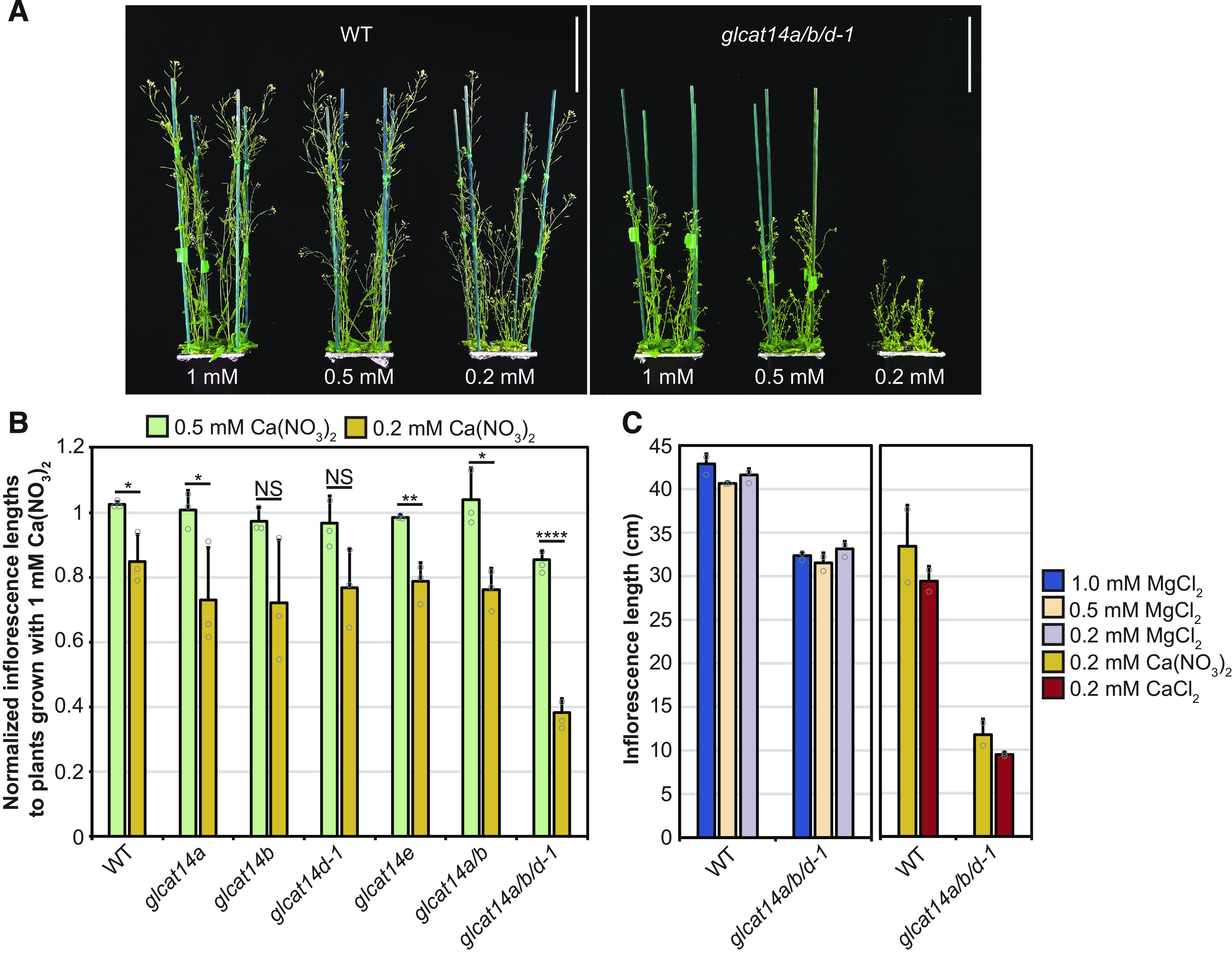
The Growth of the *glcat14a/b/d* Mutant Is Hypersensitive to Low Concentrations of Ca^2+^. **(A)** Five-week-old wild-type and *glcat14a/b/d* mutant plants grown with hydroponic solution containing 0.5 or 0.2 mM Ca(NO_3_)_2_. The mutant is hypersensitive to low Ca^2+^. Bars = 10 cm. **(B)** Inflorescence stem lengths from wild-type and *glcat14* mutant plants grown with hydroponic solution containing 1, 0.5, or 0.2 mM Ca(NO_3_)_2_. Data are normalized to inflorescence lengths from plants grown with 1 mM Ca(NO_3_)_2_. The *glcat14a/b/d* mutant growth is hypersensitive to low Ca^2+^. The chart represents means ± sd of three biological replicates. *n* = 16 per line per replicate. The significance test compares inflorescence lengths from plants grown with 0.5 versus 0.2 mM Ca(NO_3_)_2_. Asterisks indicate significant differences between mutants and the wild type defined by one-way ANOVA followed by Tukey’s multiple comparison test: *, P < 0.05; **, P < 0.01; and ****, P < 0.0001. NS, not significant. **(C)** Inflorescence lengths of 5-week-old wild-type and *glcat14a/b/d* plants in control experiments to identify any effect with the alternative divalent cation Mg^2+^ and to identify any effects of differing NO_3_^−^ concentrations. The *glcat14a/b/d* mutant was not sensitive to lowered Mg^2+^ when grown in hydroponic solutions containing 1, 0.5, or 0.2 mM MgCl_2_ and constant 2 mM Ca(NO_3_)_2_. The concentration of MgCl_2_ was 2 mM in the hydroponic solutions comparing growth on 0.2 mM Ca(NO_3_)_2_ versus 0.2 mM CaCl_2_. The graph represents means ± sd of two biological replicates. *n* = 16 per line per replicate. Wild-type samples shown were grown alongside each set of mutants.

The observed growth and developmental defects in the mutants might result from reduced Ca^2+^ availability arising from defective Ca^2+^ interaction with the [Me]GlcA-deficient AGPs. To investigate any altered sensitivity to Ca^2+^ concentration, the single, double, and triple *glcat14* mutants were grown using hydroponic medium with a controlled concentration of Ca^2+^ using Ca(NO_3_)_2_ or CaCl_2_, which affects the apoplastic [Ca^2+^] (Conn et al., 2011). The inflorescence stems of 5-week-old plants were measured to quantify the influence of Ca^2+^ concentration on plant growth (Figures 7A and 7B). A control experiment was performed in which the concentration of the divalent ion Mg^2+^ was changed while keeping the Ca^2+^ concentration in the growth medium unchanged. No growth changes were seen when the concentration of Mg^2+^ was changed (Figure 7C). In contrast, the inflorescence stem length of *glcat14a/b/d-1* triple mutants was more sensitive to low concentrations of Ca^2+^ compared with wild-type, single mutant, and *glcat14a/b* double mutant plants. This effect was seen when the concentration of Ca^2+^ was reduced using Ca(NO_3_)_2_ (Figures 7A and 7B) or CaCl_2_ (Figure 7C), indicating that the effect is specifically due to Ca^2+^ concentration.

**Figure 8.**
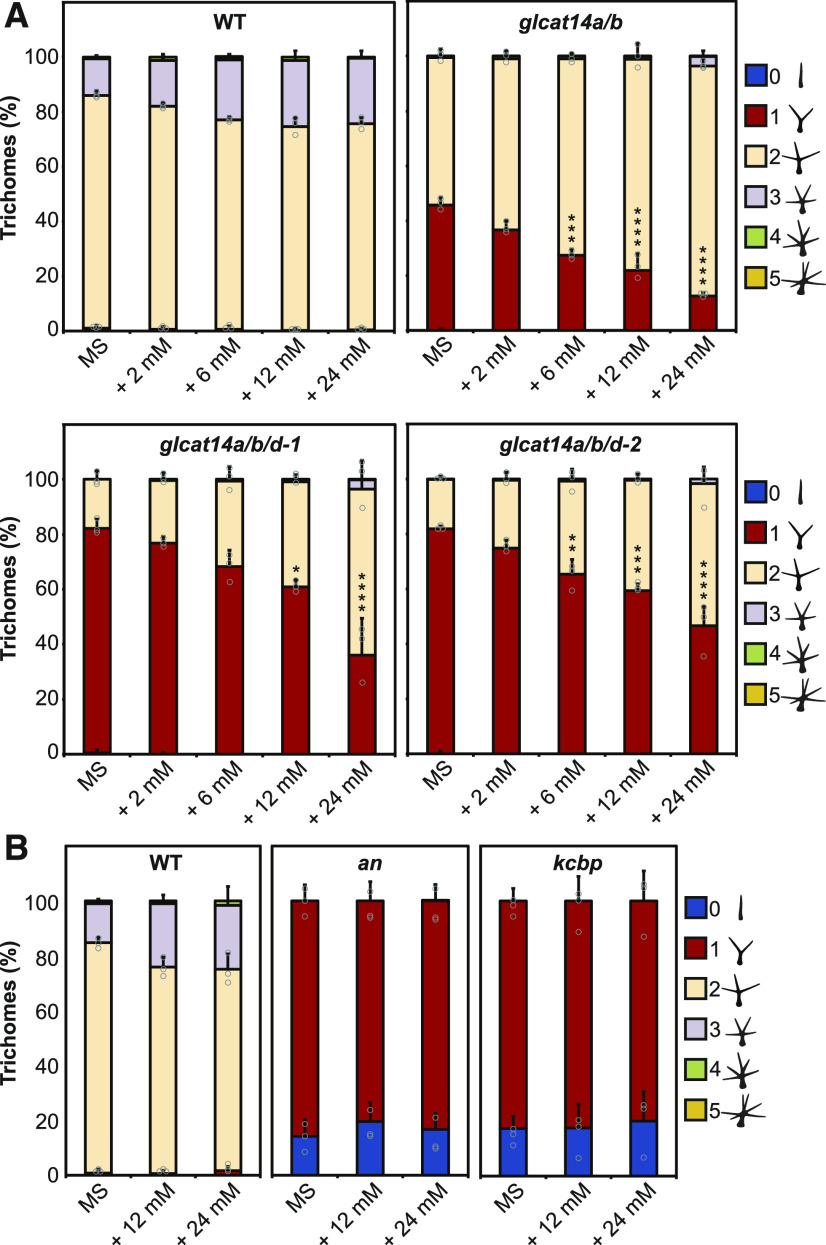
The Trichome-Branching Phenotype of *glcat14a/b* and *glcat14a/b/d* Is Suppressed by Ca^2+^. **(A)** Percentage of third true leaf trichome branching from 15-d-old wild-type, *glcat14a/b*, and *glcat14a/b/d* plants grown on basal MS medium and MS medium supplemented with 2, 6, 12, or 24 mM CaCl_2_. **(B)** Percentage of third true leaf trichome branching from 15-d-old wild-type, *an*, and *kcbp* plants grown on basal MS medium and MS medium supplemented with 12 or 24 mM CaCl_2_. The charts represent means ± sd of three biological replicates. *n* = 10 leaves per line per replicate. Data points are shown for trichomes with one and two branching points **(A)**. Only for *an* and *kcbp*, data points from trichomes with null and one branching point are shown **(B)**. Asterisks indicate significant differences of trichomes with one branching point between MS and increased CaCl_2_ as defined by one-way ANOVA followed by Tukey’s multiple comparison test: *, P < 0.05; **, P < 0.01; ***, P < 0.001; and ****, P < 0.0001.

We hypothesized that the trichome-branching phenotype of the AG glucuronidation mutants might also be influenced by limited Ca^2+^ availability in the cell-surface apoplast. Therefore, the *glcat14* mutants were grown on basal MS medium, which contains 2.99 mM CaCl_2_, and MS medium supplemented with additional 2, 6, 12, and 24 mM CaCl_2_. The trichome branching points from the third true leaves were quantified in the wild type, *glcat14a/b* double mutants, and *glcat14a/b/d-1* and *glcat14a/b/d-2* triple mutants. When the growth medium was supplemented with Ca^2+^, the trichome-branching phenotype in the *glcat14a/b* double and *glcat14a/b/d* triple mutants was overcome in a Ca^2+^ concentration-dependent manner (Figure 8A). To determine whether the phenotype in trichome mutants unrelated to AGPs is also suppressed by the addition of Ca^2+^, two trichome mutants with reduced branching, *angustifolia* (*an*; Luo and Oppenheimer, 1999) and *kinesin-like calmodulin binding protein* (*kcbp*; Oppenheimer et al., 1997), were grown on MS medium supplemented with Ca^2+^ (Figure 8B). The function of AN and KCBP (also known as ZWICHEL) has been described to be central for trichome branching initiation (Smith and Oppenheimer, 2005). In contrast to the AG *glcat14* mutants, no suppression of the branching phenotype was identified for *an* or *kcbp*, suggesting that Ca^2+^ sensitivity is not common among trichome mutants with reduced branching.

**Figure 9.**
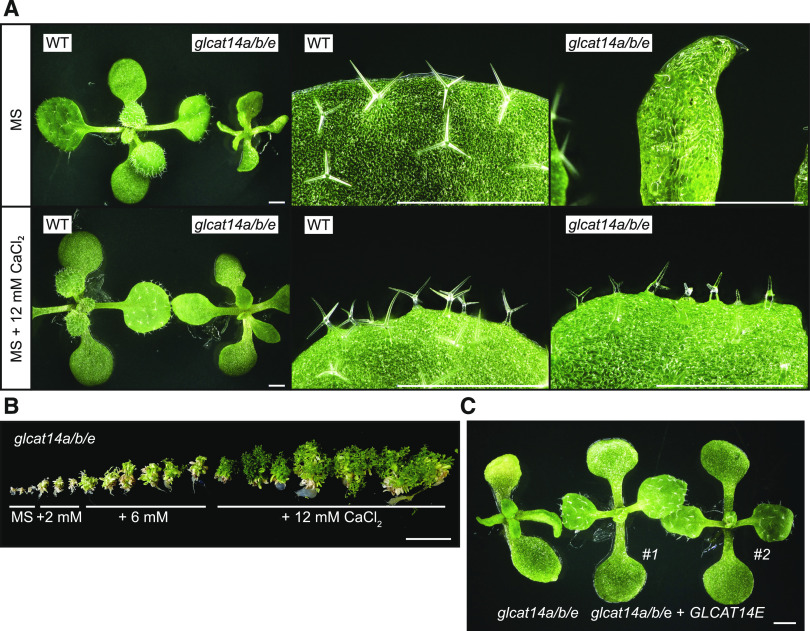
The Severe Growth Phenotype of *glcat14a/b/e* Is Partially Suppressed by Ca^2+^. **(A)** Comparison of seedling and third true leaf growth from 15-d-old wild-type and *glcat14a/b/e* plants grown on basal MS medium and MS medium supplemented with 12 mM CaCl_2_. Bars = 1 mm. **(B)** Four-month-old *glcat14a/b/e* plants grown in vitro on basal MS medium and MS medium supplemented with 2, 6, and 12 mM CaCl_2_. Bar = 5 cm. **(C)** Genetic complementation of *glcat14a/b/e*. Twelve-day-old seedlings from *GLCAT14E*-complemented lines #1 and #2 show normal leaf expansion. Bar = 1 mm.

In a third test of Ca^2+^ involvement in the AG glucuronidation mutant phenotypes, we investigated whether the severe growth phenotype of *glcat14a/b/e* triple mutants was Ca^2+^ sensitive. Indeed, *glcat14a/b/e* seedlings grown on MS medium supplemented with 12 mM CaCl_2_ grew larger than seedlings grown on basal MS medium. The *glcat14a/b/e* cotyledons were also bigger and less curved than cotyledons from seedlings grown on basal MS (Figures 9A and 9B). Similarly, true leaves and trichomes from *glcat14a/b/e* did not expand, and their trichomes lacked a defined shape when grown on basal MS medium. In contrast, when grown on the CaCl_2_-supplemented medium, the true leaves expanded and trichomes developed defined branches. However, the growth was not fully restored to wild-type levels (Figure 9B). Because only one mutant allele for *GLCAT14E* was available, segregating *glcat14a*^*+−*^*/b/e* plants were genetically complemented. The progeny of two independent lines of *glcat14a*^*+−*^*/b/e* expressing *GLCAT14E*_*pro*_:*GLCAT14E-GFP* did not show the characteristic weak phenotype of *glcat14a/b/e* (Figure 9C). The growth phenotype was instead recovered, true leaves expanded, and the trichomes showed similar branching to *glcat14a/b* double mutant seedlings. Thus, the genetic complementation of *GLCAT14E* confirmed that the mutagenesis of this gene is involved in the phenotypes observed in *glcat14a/b/e* triple mutants.

**Figure 10.**
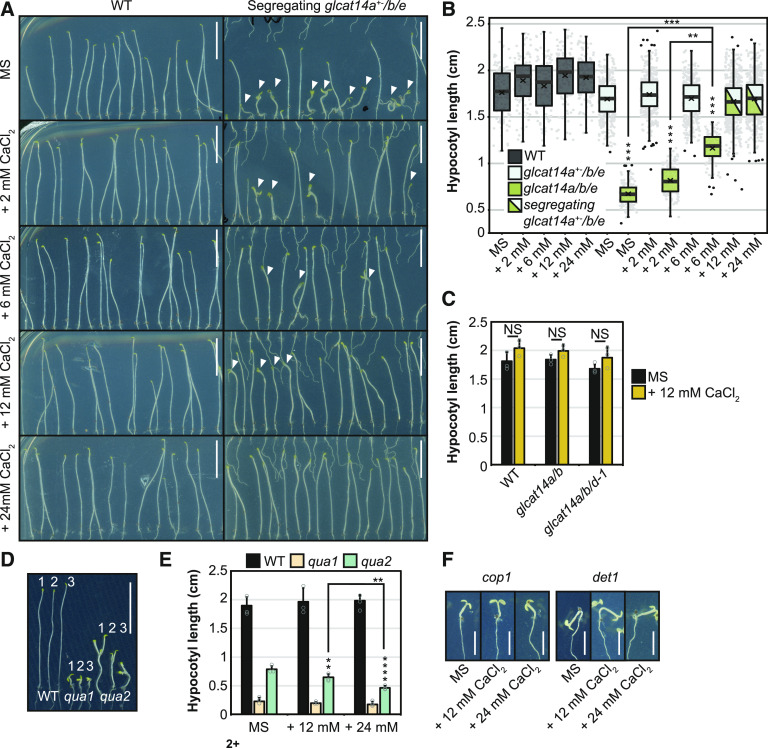
Suppression by Ca^2+^ of the Severe Etiolated Hypocotyl Phenotype of *glcat14a/b/e*. **(A)** Nine-day-old dark-grown seedlings from wild-type and segregating *glcat14a*^*+−*^*/b/e* plants grown on basal MS medium and MS medium supplemented with 2, 6, 12, or 24 mM CaCl_2_. Arrowheads indicate *glcat14a/b/e* triple mutants. Bars = 0.5 cm. **(B)** Box-plot representation of the lengths of hypocotyls from **(A)**. Gray dots represent the value of single measurements, and black dots are outliers. The cross represents the mean value of three biological replicates, and the horizontal line represents the median. Hypocotyls from *glcat14a*^*+−*^*/b/e* and *glcat14a/b/e* were not fully discernible when grown on MS medium supplemented with 12 and 24 mM CaCl_2_ and were therefore not used for statistical analysis. *n* = 50 wild-type, 180 *glcat14a*^*+−*^*/b/e*, and 50 *glcat14a/b/e* hypocotyls per treatment per replicate. **(C)** Hypocotyl lengths of 9-d-old dark-grown wild-type, *glcat14a/b*, and *glcat14a/b/d* seedlings grown on basal MS medium and MS medium supplemented with 12 mM CaCl_2_. *n* = 120 per line per replicate. **(D)** Nine-day-old dark-grown wild-type, *qua1*, and *qua2* seedlings grown on basal MS medium (1) and MS medium supplemented with 12 mM (2) or 24 mM (3) CaCl_2_. Bar = 0.5 cm. **(E)** Quantification of the length of hypocotyls from **(D)**. Values are means ± sd of three biological replicates. *n* = 25 per line per replicate. **(F)** Nine-day-old dark-grown wild-type, *cop1*, and *det1* seedlings grown on basal MS medium and MS medium supplemented with 12 and 24 mM CaCl_2_. Bars = 0.25 cm. For **(B)**, asterisks indicate significant differences between *glcat14a*^*+−*^*/b/e* and *glcat14a/b/e* as defined by two-way ANOVA followed by Sidak’s multiple comparison test (asterisks above the boxes). Significant differences between MS medium and increased CaCl_2_ were defined by one-way ANOVA followed by Tukey’s multiple comparison test. For **(C)** and **(E)**, significant differences between lines **(C)** and treatments **(E)** were defined by one-way ANOVA followed by Tukey’s multiple comparison test or Student’s *t* test for two-sample comparisons: **, P < 0.01 and ****, P < 0.0001. NS, not significant.

To test whether the partially deetiolated phenotype of *glcat14a/b/e* dark-grown seedlings was Ca^2+^ sensitive, seedlings were grown on MS medium supplemented with Ca^2+^. Hypocotyl length from the wild type and segregating *glcat14a*^*+−*^*/b/e* heterozygous mutants remained unchanged when grown on basal MS medium and MS medium supplemented with 2, 6, 12, and 24 mM CaCl_2_ (Figures 10A and 10B). Similarly, *glcat14a/b* and *glcat14a/b/d-1* hypocotyls were not sensitive to the increased concentration of Ca^2+^ (Figure 10C). In contrast, the hypocotyls from *glcat14a/b/e* elongated in a [Ca^2+^]-dependent manner (Figures 10A and 10B). Furthermore, the partially deetiolated cotyledon phenotype of *glcat14a/b/e* was fully suppressed on medium supplemented with 24 mM CaCl_2_ (Figure 10A).

In addition to AGPs, homogalacturonan is able to bind calcium in the cell wall (Willats et al., 2001). The GlcA residue of AGPs is a covalent link between an AGP and pectin in APAP1 (Tan et al., 2013). Thus, if GlcA residues in molecules such as APAP1 are important for pectin synthesis or organization in the wall, then the AG *glcat14* mutants might have defective pectin. Although cell wall analysis suggested that there is no change in homogalacturonan quantity (Supplemental Figure 3), we nevertheless investigated whether the growth of mutants deficient in pectin synthesis responds to changes in the concentration of Ca^2+^ in the growth medium. Quasimodo1 (QUA1), also known as Galacturonosyltransferase8, is a putative GalA transferase (Bouton et al., 2002; Caffall et al., 2009), and QUA2 is a putative methyltransferase in pectic homogalacturonan synthesis (Mouille et al., 2007; Mohnen, 2008). Unlike the *glcat14* mutants, the partially deetiolated hypocotyl growth phenotypes of dark-grown *qua1* and *qua2* were not suppressed by the addition of Ca^2+^ (Figures 10D and 10E). In contrast, the elongation of *qua2* hypocotyls was reduced by the high concentration of Ca^2+^ in the growth medium. Therefore, the hypocotyl phenotypes of the *glcat14* mutants are unlikely due to pectin deficiency or defective Ca^2+^ binding by pectin.

We investigated any Ca^2+^ concentration influence on the deetiolated phenotype of mutants in the photomorphogenic repressors COP1 (Deng et al., 1992) and DET1 (Pepper et al., 1994). However, no suppression of the deetiolated phenotype was observed when *cop1* and *det1* were grown on MS medium supplemented with Ca^2+^ (Figure 10F). Therefore, the suppression of the deetiolation phenotype by increasing Ca^2+^ concentration appears to be a feature specifically associated with the *glcat14* mutants.

### Intracellular Calcium Transients Are Abnormal in AG Glucuronidation Mutants

**Figure 11.**
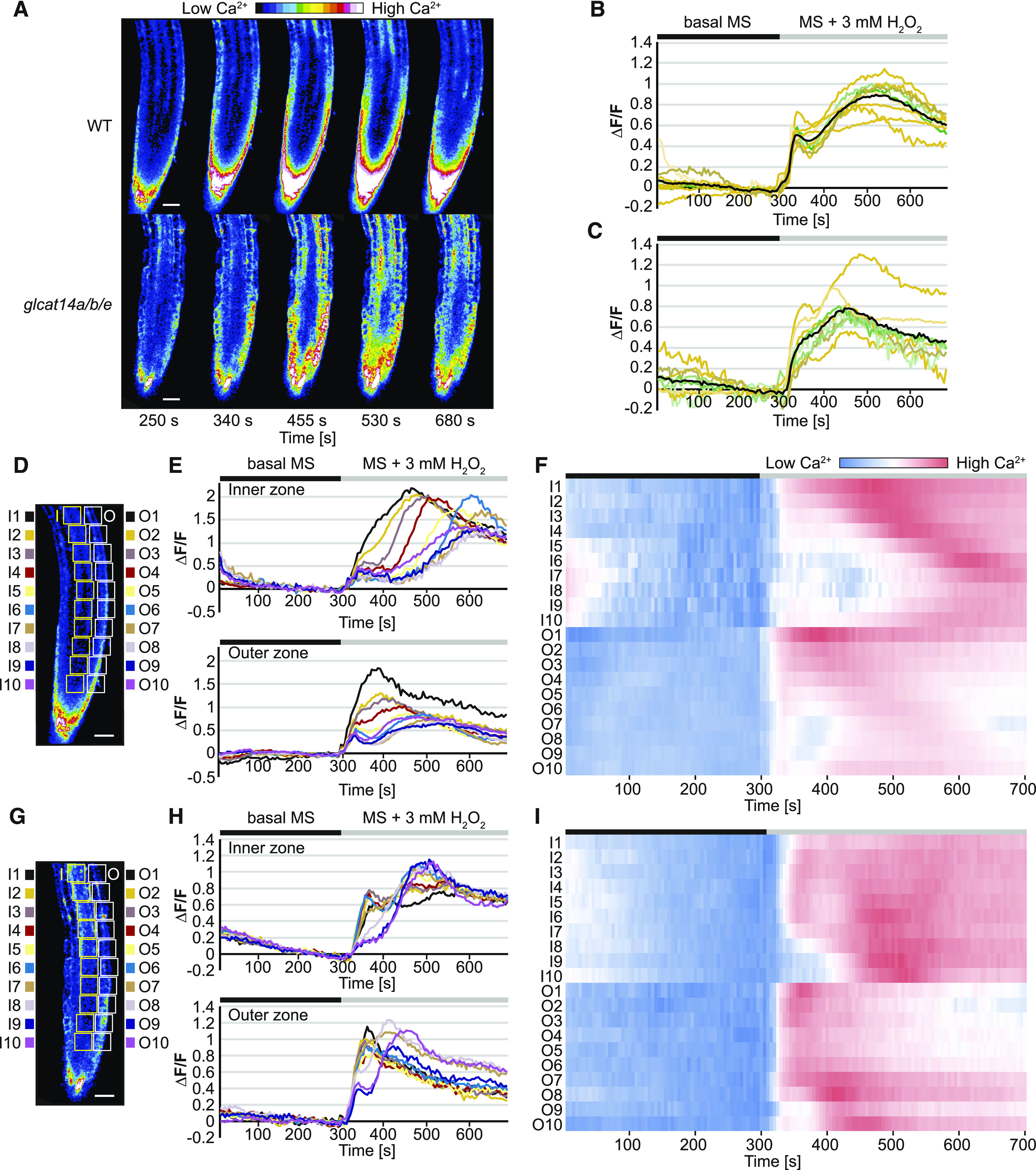
H_2_O_2_-Induced [Ca^2+^]_cyt_ Signals Are Perturbed in *glcat14a/b/e* Roots. **(A)** [Ca^2+^]_cyt_-dependent R-GECO1 fluorescence signal in response to 3 mM H_2_O_2_ in 4-d-old wild-type and *glcat14a/b/e* mutant seedlings. The H_2_O_2_ treatment was applied at ∼300 s. The images are still frames from Supplemental Movie 1 for the wild-type roots and Supplemental Movie 2 for *glcat14a/b/e* roots. **(B)** and **(C)** Normalized R-GECO1 fluorescence intensities (∆F/F) of [Ca^2+^]_cyt_ transients induced by 3 mM H_2_O_2_ in wild-type **(B)** and *glcat14a/b/e* **(C)** roots. The orange and green lines are single biological replicates. The black lines represent the mean values of the biological replicates. *n* = 9 for the wild type and *n* = 8 for *glcat14a/b/e*. **(D)** to **(I)** Spatiotemporal analysis of H_2_O_2_-induced [Ca^2+^]_cyt_ transients from **(B)** and **(C)**. Consecutive ROIs were selected along the inner (I) and outer (O) zones of the roots from the wild type (**[D]** to **[F]**) and *glcat14a/b/e* mutants (**[G]** to **[I]**). The normalized values of R-GECO1 fluorescence intensities from each ROI are represented by line graphs (**[E]** and **[H]**). Heat maps represent normalized values across ROIs at the inner and outer zones of the roots (**[F]** and **[I]**). The bars above the graphs indicate the adaptation time with basal MS (black) and the time of perfusion with the treatment (gray). Bars in **(D)** and **(G)** = 30 μm.

It has been proposed that AGPs can act as Ca^2+^ capacitors based on the idea that AGPs bind and release Ca^2+^ ions at the extracellular side of the plasma membrane (Lamport and Várnai, 2013). According to the hypothesis, this interaction can generate a source of Ca^2+^ required for the influx of Ca^2+^ from the cell-surface apoplast to the cytosol. To investigate whether AGs influence cytosolic Ca^2+^ dynamics, we imaged roots using the stably expressed sensor R-GECO1 (Keinath et al., 2015). We considered that the *glcat14a/b/e* triple mutants were most likely to show clear changes in cytosolic Ca^2+^ dynamics. Although we could not measure glucuronidation in the roots of *glcat14a/b/e* mature plants because of the severe growth phenotype, glucuronidation of root AG from *glcat14a/b* is reduced by 70% (Figure 3). Moreover, AG from *glcat14a/b/e* root-derived callus is 85% deficient in glucuronidation (Figure 4). GLCAT14A, -B, and -E are expressed in a range of cells within roots (Supplemental Figures 1 and 4), indicating a probable widespread reduction in glucuronidation. The application of exogenous H_2_O_2_ induces an influx of apoplastic Ca^2+^ into the cytosol in Arabidopsis roots (Richards et al., 2014). Therefore, a solution of H_2_O_2_ was applied to roots of 4-d-old seedlings, where the growth of wild-type and mutant plants was similar, and the [Ca^2+^]_cyt_ signature was recorded (Figure 11; Supplemental Movies 1 and 2). After recording time-lapse images for 320 s of adaptation, the treatment was applied. For the first 120 s after the application of the treatment, [Ca^2+^]_cyt_ from wild-type and *glcat14a/b/e* roots, averaged through the whole field of view, increased in a similar manner, demonstrating the competence of R-GECO1-transformed lines to respond to H_2_O_2_ (Figure 11A). However, the patterns of the [Ca^2+^]_cyt_ signatures significantly diverged after 455 s. *glcat14a/b/e* plateaued at 455 s, while the wild type reached a higher plateau at 530 s (Figures 11B and 11C). This indicates that the glucuronidation deficiency of the AG influences intracellular Ca^2+^ transient signals.

Analysis of time-lapse images revealed that Ca^2+^ waves along the root induced by H_2_O_2_ moved differently in *glcat14a/b/e* compared with the wild type, especially at the endodermis and the stele. We therefore explored any differences in spatiotemporal Ca^2+^ signatures induced by H_2_O_2_ in *glcat14a/b/e* mutant and wild-type roots. A series of consecutive regions of interest (ROIs) toward the tip of the root were used to quantify the Ca^2+^ signature at the inner zone (including endodermis, pericycle, and stele) and at the outer zone (including epidermis and cortex; Figures 11D and 11G). In wild-type roots, the increase of [Ca^2+^]_cyt_ occurs in a chronological and spatial manner reminiscent of a wave moving mainly along the inner zone and also, to a lesser degree, at the outer zone (Figures 11E and 11F; Supplemental Figure 5) toward the root tip. These [Ca^2+^]_cyt_ waves were not present at the inner or the outer zone of *glcat14a/b/e* roots (Figures 11H and 11I; Supplemental Figure 6). The less organized Ca^2+^ signature in *glcat14a/b/e* roots indicates a disruption in Ca^2+^ signaling along specific root tissues and cell types.

## DISCUSSION

In this study, we established that the interaction between AG polysaccharides and Ca^2+^ is important for several aspects of plant development and provide evidence that the cell-surface AGPs provide a source of apoplastic Ca^2+^ for its signaling. We identified and characterized the role of four Arabidopsis GlcATs that add GlcA to AG polysaccharides, GLCAT14A, GLCAT14B, GLCAT14D, and GLCAT14E. AGs from the mutant plants had reduced glucuronidation. Defective AGs bound less Ca^2+^ in vitro, and mutant plants showed multiple growth and developmental deficiencies that were suppressed by increasing Ca^2+^ in the growth medium. The loss of cell-surface AG glucuronidation also led to altered intracellular Ca^2+^ signals in response to H_2_O_2_. These results suggest that the abundant AGPs at the cell surface may provide apoplastic Ca^2+^ for influx into the cell.

### GLCAT14 Enzymes Glucuronidate Specific Structures in AG in Leaves and Roots

To study the role of [Me]GlcA on AGs, we first aimed to identify and characterize the GLCAT14 enzymes that transfer GlcA to AG polysaccharides in leaves and roots. Previously, a small reduction in glucuronidation was found in *glcat14a* mutant root AGs, indicating the existence of other GlcATs with similar specificity (Knoch et al., 2013). Our results confirm that GLCAT14B is a GlcAT for AG and show that GLCAT14A and GLCAT14B are partly redundant to each other. PACE analysis of AG extracts from *glcat14a*, *glcat14b*, and *glcat14a/b* mutants indicated that both enzymes are responsible for most of the glucuronidation directly of the β-(1→3)-galactan backbone and the short single residue β-(1→6)-linked galactan side chains of leaf and root AG polysaccharides. Our observations support the conclusions from in vitro experiments that GLCAT14A and GLCAT14B transfer GlcA onto both β-(1→3)- and β-(1→6)-galactans (Dilokpimol and Geshi, 2014). We also studied GLCAT14D and GLCAT14E as further candidates for glucuronidation of leaf and root AGs. The analysis of triple mutants *glcat14a/b/d* and *glcat14a/b/e* showed that these two GLCAT14 enzymes also glucuronidate AG. In contrast to GLCAT14A and GLCAT14B, GLCAT14D and GLCAT14E contribute GlcA preferentially to longer side chains of AG.

A substrate preference in vitro of AG GlcATs from radish (*Raphanus sativus*) roots was reported for β-(1→6)-galactooligosaccharides of DP3 or longer (Endo et al., 2013), consistent with our observations that galactan side chain lengths influence the activity of AG GlcATs. Besides the substrate length, the GlcAT activity will be influenced by other factors in vivo. First, AGs are highly branched and complex molecules decorated by different residues. Before glucuronidation, potential galactan substrates could be substituted possibly by Ara, which might also be decorated with other sugars (Tryfona et al., 2012). It is likely that the GlcA activity towards substituted galactans is different than towards non-substituted galactans. Second, GlcATs may also be specific for different AG molecules and parts of the AG molecule. This can possibly be determined by the substrate position and neighboring polysaccharides within the same AG molecule. Third, the GlcAT specificity can also be defined by its tissue- and cell-specific expression, supported by the identification of specific AGPs in specialized cell types (Coimbra et al., 2009). In spite of these yet unexplored elements, the current evidence suggests that the characterized members of clade B4.i (Dilokpimol and Geshi, 2014) and clade B5 glucuronidate the β-(1→3)-galactan backbone and the first Gal from the β-(1→6)-linked galactan side chain (Knoch et al., 2013), whereas clade B6 and clade B7 members glucuronidate β-(1→6)-linked galactans longer than Gal_2_. There are several further candidate GlcATs encoded in the Arabidopsis genome, and it will be interesting to determine whether these transfer GlcA onto specific AGPs, specific structures of AG polysaccharides, or are expressed in restricted cell types.

The analysis of AGs from *glcat14* mutants did not support the hypothesis that GlcA terminates the elongation of β-(1→6)-galactan side chains (Knoch et al., 2013), because galactan chain lengths and AG galactose content were not increased. Our results also did not show substantial changes in pectin quantity in mutant plants. This suggests that in the leaves and callus from the *glcat14* mutants analyzed here, any pectin-AGP covalent interactions through GlcA on AG, such as those reported in the APAP1 proteoglycan from cell cultures (Tan et al., 2013), were either not affected or not important for pectin biosynthesis.

### AG Glucuronidation and Plant Development

The generation of *glcat14* triple mutants with low levels of AG glucuronidation resulted in plants with multiple growth deficiencies. A severe reduction of glucuronidation on AGs in *glcat14a/b/d* reduced the inflorescence stem length. Mutants in the AGP Hyp *O*-galactosyltransferases, which initiate the AG glycans, also show reduced stem length (Ogawa-Ohnishi and Matsubayashi, 2015). The recent observation that knockout *glcat14a/b*, *glcat14b/c*, and *glcat14a/b/c* mutants have shorter inflorescences (Zhang et al., 2020) supports our finding that AGP glucuronidation is important for plant development. The triple *glcat14a/b/e* mutant showed very limited growth and was unable to produce progeny. While all four GlcATs are expressed in dark-grown seedlings, only the *glcat14a/b/e* triple mutants showed differences in the development of hypocotyls, being smaller than the wild type and showing a mild deetiolated phenotype. *glcat14* mutants *glcat14a/b* and *glcat14a/b/d* had reduced trichome branching. The reduction of trichome branching in *glcat14a/b* mutants was recently reported in another study (Zhang et al., 2020). The reduction of trichome branching together with the crooked trichome, seedlings, and characteristic etiolated hypocotyl phenotype of the *glcat14a/b/e* triple mutants suggest that glucuronidation of AGs is essential for cell shape formation and expansion. Cell expansion is regarded as a key process for trichome development and branching formation (Hülskamp, 2004; Smith and Oppenheimer, 2005).

### Calcium Binding to AG Polysaccharides and Plant Development

The large reduction of glucuronidation of AGs from *glcat14* mutants provided the opportunity to investigate in vitro and in vivo the AGP-Ca^2+^ capacitor hypothesis (Lamport and Várnai, 2013). Indeed, in vitro, the AGPs from *glcat14a/b/d-1* bound nearly 80% less Ca^2+^ than wild-type AGPs, consistent with the magnitude of reduction in AG glucuronidation, since one Ca^2+^ ion coordinates two [Me]GlcA residues. The developmental phenotypes of *glcat14* mutants are likely related to the reduction of Ca^2+^ binding capacity of the AG from AGPs, because many of these phenotypes were hypersensitive to a decreased concentration of Ca^2+^ or suppressed by an increased concentration of Ca^2+^ in the growth medium. For example, the *glcat14a/b/d* triple mutant inflorescence stem growth was hypersensitive to a low concentration of Ca^2+^ in the growth medium. Moreover, the reduced branching trichome phenotype in *glcat14a/b* double and *glcat14a/b/d* triple mutants, and the growth and short etiolated hypocotyl phenotypes of *glcat14a/b/e*, were suppressed by increasing Ca^2+^ concentration. Some FERONIA receptor-like protein kinase mutant *fer* phenotypes can be suppressed by increased Ca^2+^, perhaps by increasing Ca^2+^ binding by pectin (Feng et al., 2018). However, we believe that the phenotypes are not directly connected to pectin defects in Ca^2+^ binding because we saw no change in pectic monosaccharide composition of the walls. Furthermore, increased Ca^2+^ in growth medium did not suppress, or indeed further reduced, the short-hypocotyl phenotype of dark-grown pectin mutants *qua1* and *qua2*. Together, these findings suggests that the *glcat14* mutant phenotypes do not arise from pectin defects or signaling of pectin defects (Verger et al. 2016), but changes in pectin structure or a cell wall integrity response cannot be excluded.

The phenotype of reduced AG glucuronidation is distinct from that of Ca^2+^ transport mutants, such as null mutants of the vacuolar Ca^2+^/H^+^ antiporter CATION EXCHANGER1 (CAX1) and CAX3 and the plasma membrane Ca^2+^ channel CYCLIC NUCLEOTIDE-GATED CHANNEL2 (CNGC2). In growth conditions with low concentration of Ca^2+^ (0.1 mM), *cax1cax3* and *cngc2* grow as wild-type plants, whereas in the presence of additional Ca^2+^ (10 mM), *cax1cax3* and *cngc2* are dwarfed (Chan et al., 2003; Cheng et al., 2005; Wang et al., 2017). This phenotype was suggested to be caused by an overaccumulation of Ca^2+^ at the apoplast (Wang et al., 2017). In contrast to these transport mutants, a higher concentration of Ca^2+^ allowed better plant growth in *glcat14* triple mutants.

A high concentration of Ca^2+^ does not suppress the reduced branching trichome phenotype in *an* and *kcbp* trichome mutants (Folkers et al., 1997; Oppenheimer et al., 1997). Although their phenotype is similar to that of the AG glucuronidation mutants, these are mutants in genes unrelated to AGPs or their synthesis. Interestingly, the function of KCBP has been reported to be regulated by Ca^2+^ and Ca^2+^ binding proteins in vitro (Reddy et al., 2004; Vinogradova et al., 2009). Furthermore, the trichome branching initiation model suggests that AN interacts with the kinesin KCBP at the cell cortex of the nascent branching point to facilitate the localized delivery of Golgi vesicles (Smith and Oppenheimer, 2005). Considering the hypothesis of the cell-surface AGP-Ca^2+^ capacitor, an increase in the Ca^2+^ concentration by release from AGPs at the extracellular side of the trichome branching point may promote a local Ca^2+^ influx enabling subsequent branching initiation.

The AGP-Ca^2+^ interaction at the cell-surface apoplast has been hypothesized to drive a number of cellular processes, including pollen tube elongation (Lamport et al., 2014, 2018a), but these processes were not studied here. However, *unfertilized embryo sac7* and *tube growth defective12*, both mutants in *AT3G03690*, a member of the GT14 family, were identified in two independent screening assays for defects in fertilization and pollen tube elongation (Pagnussat et al., 2005; Boavida et al., 2009). Deficiencies in pollen germination and development have recently been reported in *glcat14abc* compound mutants (Zhang et al., 2020). AT3G03690 is likely to be an AG GlcAT, and so further studies on the effect of these mutations on pollen growth may provide additional evidence for the biological function of the AGP-Ca^2+^ interaction at the pollen cell surface.

### Calcium Waves Are Abnormal in AG Glucuronidation Mutants

To investigate the significance of the AGP-Ca^2+^ interaction for Ca^2+^ signaling, [Ca^2+^]_cyt_ was analyzed in wild-type and *glcat14a/b/e* roots expressing the Ca^2+^ reporter R-GECO1. The induction of [Ca^2+^]_cyt_ transients with H_2_O_2_ revealed a significantly altered [Ca^2+^]_cyt_ signature in *glcat14a/b/e* roots (Figure 11). A similar changed [Ca^2+^]_cyt_ response to H_2_O_2_ was reported in roots of Arabidopsis *annexin1* (*ann1*) mutants (Richards et al., 2014). ANN1 is a reactive oxygen species (ROS)-activated Ca^2+^ channel that allows apoplastic Ca^2+^ influx (Laohavisit et al., 2012; Richards et al., 2014). Similarly, leaves from null mutants of the CNGC2-CNGC4 plasma membrane Ca^2+^ channel had a deficient [Ca^2+^]_cyt_ response to H_2_O_2_ and to the bacterial flagellar peptide 22 (Tian et al., 2019). Therefore, the altered [Ca^2+^]_cyt_ signatures in *glcat14* mutants upon H_2_O_2_ elicitation are consistent with a changed influx of apoplastic Ca^2+^.

Spatiotemporal analyses revealed that [Ca^2+^]_cyt_ wave propagation was notable at the inner and outer zones of wild-type roots, but the wave was disorganized in *glcat14a/b/e* mutant roots. This suggests that glucuronidation of AGs contributes to the cell-to-cell [Ca^2+^]_cyt_ wave propagation. Oscillations in [Ca^2+^]_cyt_ have been reported to occur in close connection with extracellular pH and ROS oscillations in root hair growth (Monshausen et al., 2007, 2008). Root systemic [Ca^2+^]_cyt_ wave propagation also requires extracellular ROS production (Evans et al., 2016). Although vacuolar Ca^2+^ was shown to be important, apoplastic Ca^2+^ was suggested also to be required to generate the [Ca^2+^]_cyt_ wave (Evans et al., 2016). Since the pH at the apoplast is highly dynamic in biotic and abiotic stress (Geilfus, 2017), future studies should consider the importance of both ROS and extracellular pH changes for propagation of the [Ca^2+^]_cyt_ waves.

### Models of AGP Calcium Binding and Plant Development

**Figure 12.**
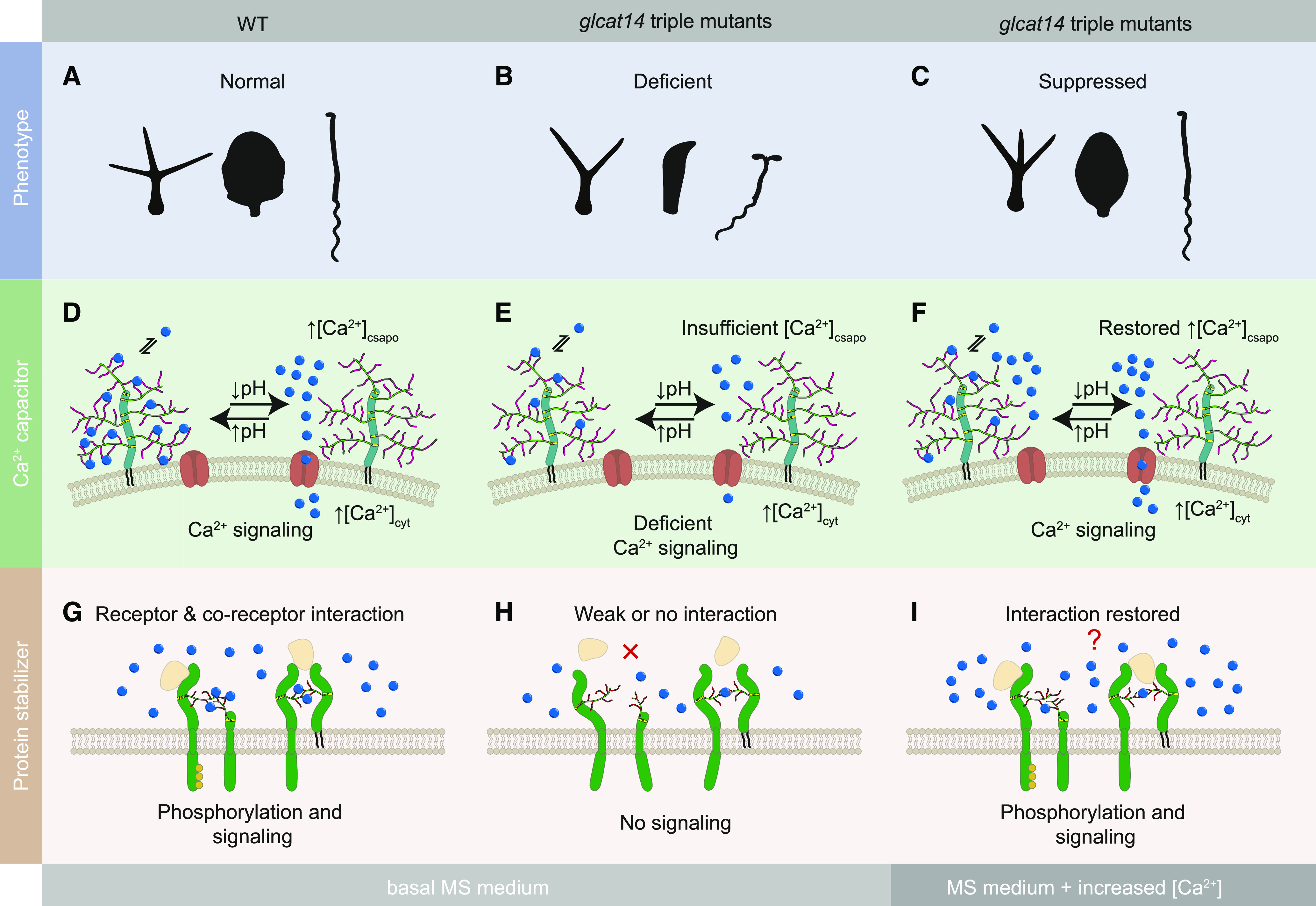
Model of the Proposed Roles for Binding of Ca^2+^ by Glucuronidated AG Polysaccharides in the Cell-Surface Apoplast. **(A)** to **(C)** Growth phenotypes were studied for the wild type **(A)** and *glcat14* mutants in the presence of basal MS medium **(B)** and MS medium supplemented with an increased concentration of Ca^2+^ **(C)**. Some growth phenotypes were suppressed when grown in medium supplemented with excess of Ca^2+^. **(D)** The reversible interaction between AGPs and Ca^2+^ is required for normal plant development. In wild-type plants, Ca^2+^ is bound by AGPs in equilibrium with [Ca^2+^]_csapo_ constituting the AGP-Ca^2+^ capacitor. When the local apoplastic space acidifies by the action of plasma membrane H^+^-ATPases, AGP [Me]GlcA becomes protonated and liberates Ca^2+^, increasing the local [Ca^2+^]_csapo_. The Ca^2+^ may be internalized via plasma membrane Ca^2+^ channels (red), driving Ca^2+^-dependent processes. The AGP-Ca^2+^ capacitor is restored as the local apoplastic pH rises. **(E)** The deficiency of glucuronidation on AGPs in *glcat14* triple mutants results in a poor binding capacity of Ca^2+^ by AGPs. This deficiency causes severe growth phenotypes, possibly resulting from insufficiency of the local [Ca^2+^]_csapo_ to activate plasma membrane Ca^2+^ channels, leading to altered Ca^2+^ signaling. **(F)** The deficient growth of *glcat14* triple mutants can be suppressed with an increase of Ca^2+^ in the growth medium. The additional Ca^2+^ in the growth medium may contribute to the required [Ca^2+^]_csapo_ threshold when the limited number of Ca^2+^ ions is liberated from the GlcA-deficient AGPs. **(G)** The interaction of glucuronidated AG polysaccharides and Ca^2+^ may be required at the cell-surface apoplast for the interactions or activity of membrane proteins such as receptor kinases or receptor-like proteins carrying arabinogalactan glycans. This interaction with Ca^2+^ may provide the correct structure for receptors and coreceptors to interact with ligands (beige). **(H)** The activity of these types of proteins is deficient in *glcat14* triple mutants. **(I)** Additional Ca^2+^ in the growth medium may restore cell-surface protein interaction and function.

The AGP-Ca^2+^ capacitor model suggests that AGPs can store Ca^2+^ and release it in a pH-dependent manner (Lamport and Várnai, 2013; Lamport et al., 2014, 2018b). Ca^2+^ could suggest a role for AGPs in Ca^2+^ buffering or homeostasis because the binding is pH dependent. It is possible that AGPs release Ca^2+^ in a stimulus-dependent manner due to changes in pH and that the increase in [Ca^2+^]_csapo_, near the plasma membrane, can affect cellular signaling. Using our genetic tools, we have been able to investigate the AGP-Ca^2+^ capacitor model and have found a role for AGPs associated with Ca^2+^ signaling. Based on our findings, we propose that the glucuronidation of AGs enables the AGP-Ca^2+^ interaction at the cell-surface apoplast, and this interaction is required for normal plant growth (Figure 12). There are several mechanisms that could require this interaction. Ca^2+^ bound to AGPs might be mobilized by transient extracellular acidification, for example by local activation of plasma membrane H^+^-ATPases, as occurs in response to auxin (Harper et al., 1989; Fendrych et al., 2016). The released Ca^2+^ might affect [Ca^2+^]_cyt_ by contributing to the influx across the plasma membrane by recruitment or activation of plasma membrane Ca^2+^ channels and/or by changing the local driving force for Ca^2+^ influx through a localized increase in external [Ca^2+^], as increases in external [Ca^2+^] activate transient and oscillatory changes in [Ca^2+^]_cyt_ (McAinsh et al., 1995). Some plant plasma membrane Ca^2+^ channels, such as CNGC2-CNGC4, are activated by an increase in external [Ca^2+^] (Tian et al., 2019). We hypothesize that the increased concentration of Ca^2+^ in the growth medium suppresses the mutant phenotypes because it partially restores the native [Ca^2+^]_csapo_ (Figures 12E and 12F) or it increases the level of binding of the Ca^2+^ to the defective AGPs (Figures 12G to 12I). In the presence of additional Ca^2+^, the scarce Ca^2+^ bound by mutant AGPs would then be released, allowing the [Ca^2+^]_csapo_ to reach the threshold of spatiotemporal Ca^2+^ concentration required for normal cellular function.

An additional or alternative role for binding Ca^2+^ may be to contribute to the stability or function of certain AGPs at the cell-surface apoplast. It was described that α-dystroglycan, a highly glycosylated mammalian receptor essential for muscle and the nervous system function, utilizes a [GlcA-Xyl]-Ca^2+^ interaction for binding with high affinity to laminin-α2 (Briggs et al., 2016). In Arabidopsis, the lysine-motif domain proteins (LYM) are GPI-anchored and predicted AG-decorated proteins (Borner et al., 2003) that participate in innate immunity. LYM1 and LYM3 form a bacterial recognition system with the receptor kinase CERK1, enabling sensitivity and resistance to bacterial infection (Willmann et al., 2011). Thus, it is possible that an interaction between ligands, receptors, or coreceptors with AG-decorated domains may be stabilized or strengthened by Ca^2+^ (Figures 12G to 12I). The AGP-Ca^2+^ interaction might also affect AGP trafficking to the cell surface. However, more investigation is required to identify the specific cellular processes and pathways dependent on the AGP-Ca^2+^ interaction.

This work demonstrates that the importance of glucuronidation of AGPs is to facilitate AG interaction with Ca^2+^. The model for AGP function as a Ca^2+^ capacitor provides an explanation for the abundance of AG-modified proteins at the cell surface (Borner et al., 2003; Lamport and Várnai, 2013). It will be important to determine when and how Ca^2+^ release from AGPs is induced during growth and development. Further study of the glucuronidation mutants will provide insight into both AGP function and the role of localized cell-surface Ca^2+^ release in plant development.

## METHODS

### Plant Material

The T-DNA insertion lines analyzed in this study were in the Arabidopsis (*Arabidopsis thaliana*) Col-0 background. The *glcat14a* (*AT5G39990*; SALK_043905; Knoch et al., 2013) and *glcat14b* (*AT5G15050*; SALK_080923) mutants were provided by Naomi Geshi (University of Copenhagen). The insertion lines *glcat14d-1* (*AT3G24040*; GK363F05.01), *glcat14d-2* (*AT3G24040*; GK_508D01), *glcat14e* (*AT3G15350*; SALK_022820), *kcbp* (*AT5G65930*; SALK_031704; Tian et al., 2015), and *an* (*AT1G01510*; SALK_026489; Chen et al., 2016) were identified using TAIR (Berardini et al., 2015) and were provided by the Nottingham Arabidopsis Stock Centre. The novel genetic material characterized or generated for this work is listed in Supplemental Data Set 1. The mutant lines *qua1* (*qua1.1*) and *qua2* (*qua2.1*) were provided by Herman Höfte (INRA-AgroParisTech). Homozygous mutants were identified by PCR genotyping (for oligonucleotide sequences, see Supplemental Table 1). To identify gene null mutants, RNA was extracted from homozygous mutant leaves using the RNAeasy Mini Kit (Qiagen). The extracted RNA was treated with DNase (RQ1 RNase-Free DNase, Promega). cDNA was generated using reverse transcriptase (SuperScript II Reverse Transcriptase, Invitrogen), and RT-qPCR was performed using oligonucleotides listed in Supplemental Table 2 and the method listed in Supplemental Table 3. For plant growth, seeds were surface sterilized and sown on solidified basal MS medium (4.4 g/L; M5519, Sigma Aldrich) containing 1.0% (w/v) Suc and 0.1% (w/v) MES, and the pH was adjusted to 5.8 using KOH and HCl. The sown seeds were stratified for 2 d at 4°C and incubated at 21°C for 15 d under white light (150 μmol m^−2^ s^−1^) with a 16-h-light/8-h-dark cycle. Seedlings were then transferred to soil (Advance M2, ICL Levington).

Plant transformation was performed using *Agrobacterium tumefaciens* strain GV3101 and the flower dipping protocol (Clough and Bent, 1998). Using the same in vitro growth conditions, 15-d-old Arabidopsis seedlings were grown on basal MS medium and MS medium supplemented with different concentrations of CaCl_2_ (C1016, Sigma Aldrich) on solidified agar plates. Similarly, for growth of hypocotyls under dark conditions, seeds were sown on solidified agar plates and stratified, and the plates were exposed for 4 h at 21°C under white light. Then, the plates were wrapped with aluminum foil and incubated in a vertical position for 9 d under darkness at 21°C.

### Phylogenetic Analysis

The CAZy database (http://www.cazy.org) was used to identify and obtain the Arabidopsis Genome Initiative gene identifier for each Arabidopsis GT14 family member (Lombard et al., 2014). Arabidopsis protein sequences of the GT14 family members were obtained using the online platform PLAZA 2.5 (Van Bel et al., 2012). Within the PLAZA website, different versions were used: *Arabidopsis thaliana* (Dicots v3.0), *Physcomitrella patens* (Dicots v3.0), *Selaginella moellendorffii* (Dicots v2.5), *Brachypodium distachyon* (Monocots v3.0), and *Populus trichocarpa* (Gymno v1.0). The catalytic sites of the GT14 sequences were aligned using the multiple sequence alignment PRANK algorithm (webPRANK; Löytynoja and Goldman, 2010). The resulting alignment is available in the Supplemental File and was then employed to construct a phylogenetic tree by using MEGA v5.2.1 software (Tamura et al., 2011). The maximum likelihood method was used to calculate the tree, and the branching robustness was calculated by bootstrapping the data set 1000 times. The resulting clades were labeled in agreement with previous reports (Ye et al., 2011; Pfeifer et al., 2020). The scale bars are in units of numbers of amino acid substitutions per site. The sequence identity matrix was done using CLC Main Workbench v6.8.2 (Qiagen). Gene expression data were extracted from the Arabidopsis eFP Browser website in 2014 using the Development RMA data set in absolute mode (Winter et al., 2007). The gene expression data for dark-grown seedlings represent transcriptomic data from hypocotyls and cotyledons grown in the absence of light (AtGenExpress Light Series from Arabidopsis eFP Browser in absolute mode; Winter et al., 2007). The cell-specific gene expression data were extracted from the Arabidopsis ePlant website in March 2020 (Waese et al., 2017) using the Tissue Specific Root eFP data set (Brady et al., 2007; Winter et al., 2007). The root image in Supplemental Figure 4 was adapted from the Arabidopsis ePlant website (Waese et al., 2017).

### Preparation of AG Extracts and AG-Specific Enzymes

AG-enriched preparations (AG extracts) were extracted from the rosette leaves of 5-week-old Arabidopsis plants. For each biological replicate, ∼48 rosette leaves were collected per line. The root AG extracts were isolated from ∼30 6-week-old plants per line grown hydroponically following previously reported protocols (Gibeaut et al., 1997). Liquid callus cultures were generated from seedling roots according to previous reports (Prime et al., 2000). Arabidopsis leaf, root, and callus AGs were extracted using previously reported protocols (Tryfona et al., 2012). AGs from wild-type and mutant plants from the same biological replicate were extracted at the same time. Different biological replicates were processed at different time frames. α-l-Arabinofuranosidase, exo-β-(1→3)-galactanase, endo-β-(1→3)-galactanase, endo-β-(1→6)-galactanase, and GUS were prepared by methods described previously (Konishi et al., 2008; Kotake et al., 2009; Takata et al., 2010; Yoshimi et al., 2017).

For some experiments, crude AG extracts were cleaned using pectin precipitation with 20 mg of copper acetate (326755, Sigma Aldrich) per mg of crude extract (Tsumuraya et al., 1988). After the removal of copper acetate from the supernatant using centrifugal filter units (Amicon 10K columns, Millipore) centrifuged at 16,160*g* for 10 min at room temperature followed by desalting columns (PD10, GE Healthcare), the AG extract was treated with 4 M KOH for 1 h. After neutralizing with acetic acid, the sample was desalted and freeze-dried. Samples were resuspended in 50 mM ammonium acetate, pH 4.5, and treated with the following pectinases provided by Novozymes: endopolygalacturonase1 (*Aspergillus aculeatus*; SWISSPROT:O74213), rhamnogalacturonan lyase (*Paenibacillus campinasensis*; SWISSPROT:A0A269W2N8), and rhamnogalacturonase A (*Aspergillus aculeatus*; SWISSPROT:Q00001). The hydrolysis with pectinases was performed for 24 h at 25°C in 15-mL tubes and 3 d at 4°C in dialysis membranes (Snakeskin 10K, Thermo Fisher Scientific) against MilliQ-grade water, which was changed three times per day. Samples were freeze-dried for storage.

### Genetic Complementation

The Golden Gate cloning system was used for cloning native promoter and coding sequences (CDSs) for the genes *AT3G15350* and *AT5G15050*. Promoter regions and CDSs were obtained from TAIR (Berardini et al., 2015). The 5′ untranslated region promoter region taken for each of the genes was 2582.0 bp (*AT3G15350*) and 915.0 bp (*AT5G15050*). The cloning was conducted following the Golden Gate DNA assembly protocol (Patron et al., 2015). Codons were optimized for the removal of the enzyme restriction sites *Bsa*I, *Bpi*I, *Esp*3I, and *Dra*III. The stop codons were also removed from CDSs. The restriction enzyme *Bsa*I was used for level 1 assembly and *Bpi*I was used for level 2 assembly. The CDSs were fused to a 3′ eGFP reporter followed by a NOS terminator. Transformants were selected using kanamycin at 50 mg/L and fluorescence microscopy to identify GFP-positive plants. Genetically complemented lines were identified in T1 seedlings.

### Enzymatic Hydrolysis and PACE Analysis

AG extracts (0.5 mg) were digested with AG-specific enzymes following previously described protocols (Tryfona et al., 2012). The products of the hydrolysis were derivatized, and the labeled carbohydrates were analyzed by PACE using previously developed protocols (Goubet et al., 2002). Control experiments were performed in the absence of enzymes in order to identify possible background unrelated to the intentionally hydrolyzed AGs. The resolved oligosaccharides from PACE were quantified using GeneTools (Syngene). The abundance of the oligosaccharides Gal_1-4_ and [Me]GlcAGal_1-4_ was quantified based on the band intensity from PACE. Then, ratios were calculated using the abundance of glucuronidated oligosaccharides over the abundance of nonglucuronidated ones of the same galactan DP. These ratios were used to compare the relative abundance of [Me]GlcA-containing oligosaccharides between the wild type and mutants.

### Acid Hydrolysis, Enzymatic Hydrolysis, and HPAEC-PAD Analysis

AG extracts (see Preparation of AG Extracts and AG-Specific Enzymes) were hydrolyzed with 2.0 M TFA for 1 h at 120°C. TFA was removed by vacuum, and the samples were resuspended in 200 μL of water. The monosaccharide analysis of acid-hydrolyzed samples was performed following protocols described previously (Tryfona et al., 2012). To determine the amounts of Ara, Gal, GlcA, and 4-*O*-Me-GlcA, AG extracts were enzymatically hydrolyzed. Samples containing 80 to 600 μg of carbohydrates were incubated with 0.12 units of exo-β-(1→3)-galactanase, 0.12 units of endo-β-(1→6)-galactanase, 0.012 units of endo-β-(1→3)-galactanase, 0.12 units of α-l-arabinofuranosidase (Megazyme), and 0.12 units of GUS. The enzymatic hydrolysis was done in 20 mM sodium acetate, pH 4.0, for 12 h at 37°C. The resulting monosaccharides and oligosaccharides were detected, and the sugar composition was determined by HPAEC-PAD using an ICS-5000+ fitted with a pulsed amperometric detector (Thermo Fisher Scientific) following protocols as described by Ishikawa et al. (2000). Monosaccharides included in the hydrolysate were separated on a CarboPac PA-1 column (4 × 250 mm; Thermo Fisher Scientific) at a flow rate of 1.0 mL/min. The elution protocol comprised a linear gradient of NaOH (20 to 35 mM, 10 min) followed by a linear gradient of sodium acetate (0 to 250 mM) in 100 mM NaOH (5.4 min) and an isocratic elution with 250 mM sodium acetate in 100 mM NaOH. For estimating total Gal, the amount of enzymatically released β-(1→6)-galactobiose was summed up as two Gal molecules for the calculation of monosaccharide composition.

### Imaging of Trichomes and Dark-Grown Seedlings

Fifteen-day-old seedlings were imaged with a digital microscope (VHX-5000, Keyence) with a fitted variable illumination attachment (VH-K20, Keyence) for improving sample illumination. For imaging trichomes, the third true leaf of 15-d-old seedlings was detached from the petiole and placed on a plate with solidified agar during imaging. The microscopy imaging was performed at the Sainsbury Laboratory, University of Cambridge. For imaging hypocotyls, 9-d-old dark-grown seedlings were scanned using a flatbed scanner (V600, Epson).

### Hydroponics for Inflorescence Growth Assay

For assaying inflorescence growth, squared pots with drainage holes (H. Smith Plastics) were used. The pots were filled in with a layer of 1 cm of vermiculite and a piece of rockwool (Cultilene). The pot’s drainage holes were filled with soft foam to avoid the loss of vermiculite while allowing water to flow through. These prepared pots were soaked in distilled water for 2 d, and the water was changed once per day. The soaked pots were covered with a layer of aluminum foil to avoid evaporation and to protect them from soil or contamination. Fifteen-day-old plants were transferred to the pots through openings in the foil. Each pot held four plants. Pots were watered with hydroponic solutions every 5th day (for composition of the solution, see Supplemental Table 4), and inflorescence length was also recorded on the same day.

### CryoSEM Imaging

The cryoSEM imaging was performed on an EVO HD15 (Zeiss) equipped with a cryoSEM preparation system (PP3010T, Quorum). True leaves from 15-d-old Arabidopsis plants were prepared according to previously published methods (Wightman et al., 2017). Liquid nitrogen-frozen samples were treated with a platinum coating of 6.0 nm, and the samples were imaged with a beam set at 6.0 kV. The samples were prepared by Ray Wightman at the Sainsbury Laboratory, University of Cambridge.

### Calcium Binding Assay

AG extracts (see Preparation of AG Extracts and AG-Specific Enzymes) from three biological replicates were used for measuring the capacity of holding Ca^2+^ ions. All AG samples were processed at the same time, and this experiment represents one technical replicate. The total concentration of carbohydrates in the AG samples was determined by the phenol-sulfuric acid method (DuBois et al., 1956). The in vitro Ca^2+^ binding assay was done following a previously reported protocol (Lamport and Várnai, 2013). Briefly, 2.5 mg of Arabidopsis pectin-free AG extracts (see Preparation of AG Extracts and AG-Specific Enzymes), gum arabic (G9752, Sigma Aldrich), and larch (*Larix* spp.) AGs (10,830, Sigma Aldrich) were resuspended in MilliQ-grade water and added to 0.5-mL centrifugal filters (Amicon 10K columns, Millipore). The samples were centrifuged at 16,160*g* for 10 min at room temperature, and the retained material was resuspended in 10 mM ammonium acetate, pH 1.5. After centrifugation at 16,160*g* for 10 min at room temperature, the retained material was resuspended in 10 mM ammonium acetate, pH 5.5. Then, a volume of 20 mM CaCl_2_ was added for a final 2 mM CaCl_2_ in 10 mM ammonium acetate, pH 5.5. The material was resuspended and incubated for 20 min at 25°C. After 20 min of incubation, the material was again resuspended and incubated for 20 min at 25°C. After centrifugation at 16,160*g* for 10 min at room temperature, the filtrate was collected for analysis. The retained material was resuspended in 10 mM ammonium acetate, pH 5.5, and recovered from the column by centrifugation at 2000*g* for 5 min at room temperature. This was followed by a second resuspension of any remaining retained material in the centrifugal unit in 10 mM ammonium acetate, pH 5.5, and recovered using centrifugation at 2000*g* for 5 min at room temperature. The recovered retained material from the two resuspensions was pooled and used for analysis. The filtrate and the recovered retained material were resuspended in 5% (v/v) nitric acid for elemental analysis. The concentration of Ca^2+^ was determined by ICP-MS. The AG Ca^2+^ binding capacity was calculated as the proportion of Ca^2+^ retained by AGs from the total amount of Ca^2+^ in the filtrate and retained material (Supplemental Data Set 2).

### Calcium Imaging

Plants were transformed with pUBQ10:R-GECO1:HSP18.2 (Keinath et al., 2015) provided by Karin Schumacher (Heidelberg University), and transformants were selected using BASTA (45,520, Sigma Aldrich). T2 seedlings from wild-type and *glcat14a/b/e* plants grown alongside each other were imaged using an upright confocal laser scanning microscope (LSM780, Zeiss) equipped with a Plan-Apochromat 20×/0.8 (Zeiss). The intensiometric Ca^2+^ sensor R-GECO1 was excited with 561 nm, and the emission was detected between 620 and 650 nm. Time-lapse images were recorded using a photomultiplier tube detector with an interval of 5 s. Four-day-old seedlings grown on basal MS medium were mounted in a perfusion system using 0.1 mm sticky-Slides I (80,168, Ibidi) following previously described protocols (Rizza et al., 2019). In the perfusion system, medium flowed from cotyledons to roots. Before acquiring the time-lapse images, mounted samples were perfused with basal MS medium for 30 min for sample adaptation.

### Image and Data Analyses

Etiolated hypocotyl lengths and trichome branching points were analyzed using FIJI (Schindelin et al., 2012). The total number of trichomes scored is listed in Supplemental Tables 5 to 7. For the analysis of Ca^2+^ dynamics, all images from confocal imaging were equally processed, the background was subtracted, Gaussian Blur was applied, and the contrast was enhanced using FIJI. The fluorescence intensity values of R-GECO1 were exported from FIJI by drawing a ROI to the entire image. The same processed images were used in the spatiotemporal analysis. Consecutive ROIs were selected along root tissues, 10 ROIs at the inner zone (includes endodermis, pericycle, and stele) and 10 at the outer zone (includes epidermis and cortex) of the root. The fluorescence intensity values were normalized using the fractional fluorescence (ΔF/F) which was calculated for each ROI from background-corrected intensity values (F − F_0_/F_0_) as described previously (Keinath et al., 2015). F_0_ represents the average fluorescence of the baseline (30 frames, 150 s) before the application of the treatment. Line graphs represent ΔF/F values for each ROI. Heat maps represent ΔF/F values for each ROI, and the color intensity was normalized independently to the highest value across ROIs at the inner zone and the outer zone of the root. Heat maps were prepared using Excel (Microsoft).

### Statistical Analysis

All tested samples were from plants grown alongside wild-type plants. A set of plants grown at the same time was considered as a biological replicate. At least three biological replicates, each of which was grown at different time frames, were used for data analysis and plotting. For statistical analysis of two samples, measurements were compared using two-tailed Student’s *t* test assuming unpaired samples and equal variance with a confidence interval (CI) of 95%. Multiple comparisons of measurements were performed using ordinary one-way ANOVA (no matching) followed by Tukey’s multiple test or ordinary two-way ANOVA followed by Sidak’s multiple comparisons test with single pooled variance. Multiple comparisons were conducted with a CI of 95%, and each P value was adjusted to account for multiplicity. Asterisks denote the value of P as follows: * P < 0.05, ** P < 0.01, *** P < 0.001, and **** P < 0.0001. For Figure 4B, a CI of 90% was used and the asterisk denotes P < 0.1. Analyses were performed in GraphPad Prism version 8.4.2 for macOS (GraphPad software), and the results can be found in Supplemental Data Set 3.

### Accession Numbers

Sequence data from this article can be found in the TAIR database under the following accession numbers: *GLCAT14A* (*AT5G39990*), *GLCAT14B* (*AT5G15050*), *GLCAT14D* (*AT3G24040*), *GLCAT14E* (*AT3G15350*), *AN* (*AT1G01510*), *KCBP* (*AT5G65930*), *QUA1* (*AT3G25140*), *QUA2* (*AT1G78240*), *COP1* (*AT2G32950*), and *DET1* (*AT4G10180*).

### Supplemental Data

**Supplemental Figure 1.** Gene expression of the GT14 family members. (Supports Figure 1)
**Supplemental Figure 2.** Percentage of amino acid identity between the members of the GT14 family. (Supports Figure 1)
**Supplemental Figure 3.** HPAEC-PAD monosaccharide composition analysis of acid-hydrolyzed AG extracts and cell wall alcohol insoluble extracts. (Supports Figure 4)
**Supplemental Figure 4.** Cell-specific gene expression of GT14 members in roots. (Supports Figure 11)
**Supplemental Figure 5.** Spatiotemporal H_2_O_2_-induced [Ca^2+^]_cyt_ signals in wild-type roots. (Supports Figure 11)
**Supplemental Figure 6.** Spatiotemporal H_2_O_2_-induced [Ca^2+^]_cyt_ signals in *glcat14a/b/e* roots. (Supports Figure 11)
**Supplemental Table 1.** Sequences of oligonucleotides used for PCR.
**Supplemental Table 2.** Sequences of oligonucleotides used for RT-qPCR.
**Supplemental Table 3.** Method for RT-qPCR.
**Supplemental Table 4.** Nutrient composition of hydroponic solutions.
**Supplemental Table 5.** Number of trichome branching points from wild type, singles, double and triple *glcat14* mutants grown on basal MS media of three biological replicates.
**Supplemental Table 6.** Number of trichome branching points from wild type, double and triple *glcat14* mutants grown on supplemented MS media with CaCl_2_ of three biological replicates.
**Supplemental Table 7.** Number of trichome branching points from *an* and *kcbp* grown on supplemented MS media with CaCl_2_ of three biological replicates.
**Supplemental Data Set 1.** Novel genetic materials.
**Supplemental Data Set 2.** Calcium binding assay data.
**Supplemental Data Set 3.** Results from statistical analyses.
**Supplemental Movie 1.** Time-course of [Ca^2+^]_cyt_-dependent R-GECO1 fluorescence in roots from wild-type seedlings in response to H_2_O_2_.
**Supplemental Movie 2.** Time-course of [Ca^2+^]_cyt_-dependent R-GECO1 fluorescence in roots from *glcat14a/b/e* seedlings in response to H_2_O_2_.
**Supplemental File.** Amino acid sequence alignment of the CAZy GT14 family.

## DIVE Curated Terms

The following phenotypic, genotypic, and functional terms are of significance to the work described in this paper:

CAX3 Gramene: AT3G51860
CAX3 Araport: AT3G51860

LYM1 Gramene: AT1G21880
LYM1 Araport: AT1G21880

AT3G15350 Gramene: AT3G15350
AT3G15350 Araport: AT3G15350

AT5G15050 Gramene: AT5G15050
AT5G15050 Araport: AT5G15050

AT3G24040 Gramene: AT3G24040
AT3G24040 Araport: AT3G24040

AT1G01510 Gramene: AT1G01510
AT1G01510 Araport: AT1G01510

QUA1 Gramene: AT3G25140
QUA1 Araport: AT3G25140

FER Gramene: AT3G51550
FER Araport: AT3G51550

AT1G03520 Gramene: AT1G03520
AT1G03520 Araport: AT1G03520

AT1G53100 Gramene: AT1G53100
AT1G53100 Araport: AT1G53100

AT1G71070 Gramene: AT1G71070
AT1G71070 Araport: AT1G71070

GLCAT14C Gramene: AT2G37585
GLCAT14C Araport: AT2G37585

AT3G03690 Gramene: AT3G03690
AT3G03690 Araport: AT3G03690

GLCAT14E Gramene: AT3G15350
GLCAT14E Araport: AT3G15350

GLCAT14D Gramene: AT3G24040
GLCAT14D Araport: AT3G24040

AT4G03340 Gramene: AT4G03340
AT4G03340 Araport: AT4G03340

AT4G27480 Gramene: AT4G27480
AT4G27480 Araport: AT4G27480

GLCAT14B Gramene: AT5G15050
GLCAT14B Araport: AT5G15050

GLCAT14A Gramene: AT5G39990
GLCAT14A Araport: AT5G39990

AN Gramene: AT1G01510
AN Araport: AT1G01510

KCBP Gramene: AT5G65930
KCBP Araport: AT5G65930

QUA2 Gramene: AT1G78240
QUA2 Araport: AT1G78240

DET1 Gramene: AT4G10180
DET1 Araport: AT4G10180

CNGC2 Gramene: AT5G15410
CNGC2 Araport: AT5G15410

CNGC4 Gramene: AT5G54250
CNGC4 Araport: AT5G54250

LYM3 Gramene: AT1G77630
LYM3 Araport: AT1G77630

CERK1 Gramene: AT3G21630
CERK1 Araport: AT3G21630

ANN1 Gramene: AT1G35720
ANN1 Araport: AT1G35720

UNE7 Gramene: AT3G03690
UNE7 Araport: AT3G03690

TGD12 Gramene: AT3G03690
TGD12 Araport: AT3G03690

CAX1 Gramene: AT2G38170
CAX1 Araport: AT2G38170

APAP1 Gramene: AT3G45230
APAP1 Araport: AT3G45230

AGM1 Gramene: AT1G27930
AGM1 Araport: AT1G27930

AGM2 Gramene: AT1G67330
AGM2 Araport: AT1G67330

GALT2 Gramene: AT4G21060
GALT2 Araport: AT4G21060

GALT3 Gramene: AT3G06440
GALT3 Araport: AT3G06440

GALT4 Gramene: AT1G27120
GALT4 Araport: AT1G27120

GALT5 Gramene: AT1G74800
GALT5 Araport: AT1G74800

GALT6 Gramene: AT5G62620
GALT6 Araport: AT5G62620

HPGT1 Gramene: AT5G53340
HPGT1 Araport: AT5G53340

HPGT2 Gramene: AT4G32120
HPGT2 Araport: AT4G32120

HPGT3 Gramene: AT2G25300
HPGT3 Araport: AT2G25300

GALT31A Gramene: AT1G32930
GALT31A Araport: AT1G32930

GALT29A Gramene: AT1G08280
GALT29A Araport: AT1G08280

UPEX1 Gramene: AT1G33430
UPEX1 Araport: AT1G33430

FUT4 Gramene: AT2G15390
FUT4 Araport: AT2G15390

FUT6 Gramene: AT1G14080
FUT6 Araport: AT1G14080

PT08G12800 Gramene: PT08G12800
PT08G12800 Araport: PT08G12800

PT10G11480 Gramene: PT10G11480
PT10G11480 Araport: PT10G11480

BD2G01620 Gramene: BD2G01620
BD2G01620 Araport: BD2G01620

BD2G35570 Gramene: BD2G35570
BD2G35570 Araport: BD2G35570

BD2G51360 Gramene: BD2G51360
BD2G51360 Araport: BD2G51360

BD1G75410 Gramene: BD1G75410
BD1G75410 Araport: BD1G75410

BD1G12030 Gramene: BD1G12030
BD1G12030 Araport: BD1G12030

BD4G00410 Gramene: BD4G00410
BD4G00410 Araport: BD4G00410

BD1G36660 Gramene: BD1G36660
BD1G36660 Araport: BD1G36660

BD2G06240 Gramene: BD2G06240
BD2G06240 Araport: BD2G06240

BD3G27240 Gramene: BD3G27240
BD3G27240 Araport: BD3G27240

BD3G14860 Gramene: BD3G14860
BD3G14860 Araport: BD3G14860

BD1G66497 Gramene: BD1G66497
BD1G66497 Araport: BD1G66497

SM00000G02860 Gramene: SM00000G02860
SM00000G02860 Araport: SM00000G02860

SM00006G05010 Gramene: SM00006G05010
SM00006G05010 Araport: SM00006G05010

PP00090G00400 Gramene: PP00090G00400
PP00090G00400 Araport: PP00090G00400

PP00149G00400 Gramene: PP00149G00400
PP00149G00400 Araport: PP00149G00400

PP00329G00090 Gramene: PP00329G00090
PP00329G00090 Araport: PP00329G00090

PP00170G00330 Gramene: PP00170G00330
PP00170G00330 Araport: PP00170G00330

PP00245G00170 Gramene: PP00245G00170
PP00245G00170 Araport: PP00245G00170

PP00338G00090 Gramene: PP00338G00090
PP00338G00090 Araport: PP00338G00090

PT08G00650 Gramene: PT08G00650
PT08G00650 Araport: PT08G00650

PT01G05380 Gramene: PT01G05380
PT01G05380 Araport: PT01G05380

PT04G13030 Gramene: PT04G13030
PT04G13030 Araport: PT04G13030

PT17G07560 Gramene: PT17G07560
PT17G07560 Araport: PT17G07560

PT09G00330 Gramene: PT09G00330
PT09G00330 Araport: PT09G00330

PT01G39950 Gramene: PT01G39950
PT01G39950 Araport: PT01G39950

PT11G11960 Gramene: PT11G11960
PT11G11960 Araport: PT11G11960

PT13G06620 Gramene: PT13G06620
PT13G06620 Araport: PT13G06620

PT13G14550 Gramene: PT13G14550
PT13G14550 Araport: PT13G14550

PT19G10460 Gramene: PT19G10460
PT19G10460 Araport: PT19G10460

PT06G26300 Gramene: PT06G26300
PT06G26300 Araport: PT06G26300
